# Comparative genome and transcriptome analyses reveal innate differences in response to host plants by two color forms of the two-spotted spider mite *Tetranychus urticae*

**DOI:** 10.1186/s12864-021-07894-7

**Published:** 2021-07-23

**Authors:** Shi-Mei Huo, Zhi-Chao Yan, Feng Zhang, Lei Chen, Jing-Tao Sun, Ary A. Hoffmann, Xiao-Yue Hong

**Affiliations:** 1 Department of Entomology, Nanjing Agricultural University, Nanjing, 210095 Jiangsu China; 2 School of BioSciences, Bio21 Institute, The University of Melbourne, Melbourne, Victoria 3010 Australia

**Keywords:** *Tetranychus urticae*, Adaptive divergence, Comparative genomics and transcriptomics, Host transfer, Transcriptional plasticity

## Abstract

**Background:**

The two-spotted spider mite, *Tetranychus urticae*, is a major agricultural pest with a cosmopolitan distribution, and its polyphagous habits provide a model for investigating herbivore-plant interactions. There are two body color forms of *T. urticae* with a different host preference. Comparative genomics and transcriptomics are used here to investigate differences in responses of the forms to host plants at the molecular level. Biological responses of the two forms sourced from multiple populations are also presented.

**Results:**

We carried out principal component analysis of transcription changes in three red and three green *T. urticae* populations feeding on their original host (common bean), and three hosts to which they were transferred: cotton, cucumber and eggplant. There were differences among the forms in gene expression regardless of their host plant. In addition, different changes in gene expression were evident in the two forms when responding to the same host transfer. We further compared biological performance among populations of the two forms after feeding on each of the four hosts. Fecundity of 2-day-old adult females showed a consistent difference between the forms after feeding on bean. We produced a 90.1-Mb genome of the red form of *T. urticae* with scaffold N50 of 12.78 Mb. Transcriptional profiles of genes associated with saliva, digestion and detoxification showed form-dependent responses to the same host and these genes also showed host-specific expression effects.

**Conclusions:**

Our research revealed that forms of *T. urticae* differ in host-determined transcription responses and that there is form-dependent plasticity in the transcriptomic responses. These differences may facilitate the extreme polyphagy shown by spider mites, although fitness differences on hosts are also influenced by population differences unrelated to color form.

**Supplementary Information:**

The online version contains supplementary material available at 10.1186/s12864-021-07894-7.

## Background

Arthropod herbivores can be specialists on a narrow range of host plants or extremely polyphagous, utilizing hundreds of hosts [1]. The adaptation of herbivores to different host plants is complicated by the complex defense strategies of plants that co-evolve with them. Apart from physical barriers developed by plants, a variety of toxic secondary metabolites produced by plants can negatively affect the feeding and digestion efficiency of herbivores [2]. Moreover, protein inhibitors in plants can be effective defense mechanisms, as confirmed in genetically modified plants [3]. Plants also release unique volatiles to interfere with the feeding of herbivores and increase the attraction of natural enemies of herbivores when plants are attacked by them [4]. In turn, herbivores can adapt to host plants in diverse ways such as through processing plant toxins, inhibiting plant defenses, or avoiding plant defenses entirely with behavioral strategies [5].

These adaptations are likely to involve changes in gene content and transcription changes in genes affecting saliva production, detoxification, metabolic processes and digestive systems of the herbivore. Secreted saliva plays an important role in modulating plant defenses, particularly through effectors, which represent important salivary constituents critical for plant-herbivore interactions [6, 7]. Metabolic processes provide resistance to plant chemicals and this interaction forms a co-evolutionary arms race between herbivores and their host plants [8]. Herbivores can defend against plant damage through a detoxification system which includes a range of enzymes [9, 10]. This detoxification system consists of three phases: the first is composed of cytochrome P450 monooxygenases (CYP450s) and carboxyl/cholinesterases (CCEs) that conduct oxidation, hydrolysis and/or reduction; the second contains glutathione S-transferases (GSTs) that carry out conjugation with hydrophilic materials; and the third includes ATP-binding cassette transporters (ABCs) that export the conjugated toxins out of the cell [9]. Digestive proteases responsible for the hydrolysis of dietary proteins are essential for herbivores to successfully process foods and absorb nutrients from their diets [11, 12]. Digestive enzymes include cysteine and serine proteases that play key roles in nutrition utilization, and cysteine peptidases that hydrolyze peptide bonds using a catalytic cysteine [13–15].

Polyphagous herbivores are adapted to a broad range of host plants often with different defense compounds. Arthropod herbivores can adapt to new host plants through various approaches, including changes in gene function and expression, as well as symbiont-assisted regulation [10, 16]. Gene family expansion and horizontal gene transfer, as well as regulation of gene expression, can be involved in herbivorous adaptation to diverse host plants [17–19]. Gene family expansion results from gene duplication, which is usually caused by unequal crossing over, segmental duplication, retrotransposon insertion or whole-genome duplication [20]. Gene duplication followed by subfunctionalization or neofunctionalization of the duplicated gene plays an important role in plant host adaptation. Comparative genomics and transcriptomics have indicated that strong expansion and neofunctionalisation of genes associated with detoxification, digestion and chemosensory functions, coupled with versatile transcriptional responses to different hosts, are involved in the extreme polyphagy of two heliothines [21]. The expansion of gene families associated with perception and detoxification have been implicated in adaptation to plant defense compounds by the cosmopolitan moth *Plutella xylostella* [22]. In addition, horizontal gene transfer from microbial species to arthropod genomes has been suggested to play important roles in the xenobiotic metabolism of herbivores, and likely aid nutrition and defense [18]. Protein products from these genes may mainly help penetration and digestion of plant cell walls, assimilation of plant nutrients, and overcoming plant defenses; for instance, genes coding for plant cell wall degrading enzymes have been laterally transferred from fungi or bacteria to herbivorous beetles to degrade complex cell wall polysaccharides [23–25]. β-Fructofuranosidases that break down plant sucrose appear to have been horizontally transferred into insect genomes from bacteria [26–30], which also participate in plant defenses of silkworm (*Bombyx mori*) [26].

Changes in gene expression can affect the range of host plants attacked by herbivores. Transcriptional plasticity, where gene expression depends on the environment and/or host, has been implicated in enhancing detoxification potential and decreasing production of plant defense compounds following exposure to novel host plants [17, 31–33]. Beneficial microbial symbionts can provide their hosts with new functions related to nutrition, digestion, and defense [34–36], allowing arthropods to feed on plants [37]. An example is the obligate bacterial symbiont (*Buchnera*) that synthesizes essential amino acids allowing aphids to feed on a phloem diet [16, 38, 39].

The two-spotted spider mite, *Tetranychus urticae*, is an extremely polyphygous pest mite with a cosmopolitan distribution, and it is reported to have a host range covering more than 1100 plants in more than 140 plant families in fields and greenhouses [17]. Two color forms exist in *T. urticae*: the green and red forms (with the latter once considered as a separate species, *Tetranychus cinnabarinus*, but the red form now considered as the same species of *T. urticae* as the green form [40, 41]). Both the red and green *T. urticae* coexist in China. Lu et al. (2017) summarized the geographical distributions of both forms from 1975 to 2014 in China based on literature reports from the SCI database and the Chinese CNKI database, and showed that the green form has expanded in recent years compared to the red form, although the two forms have varied in relative frequency in recent years [42, 43]. Additionally, collections show that both forms tend not to co-occur within the same geographical area. Population genetic investigations have indicated that genetic diversity in the red *T. urticae* is lower than in the green form [44]. Both forms have a wide host range, but the forms appear to prefer different host plants and competition between them may depend on host plant [41, 45]. For instance, the red form seems relatively better adapted to cotton [45]. Mechanisms involved are unclear, although the green form appears to have adapted to different hosts through multiple defense systems and recruitment of novel genes by the process of horizontal gene transfer (HGT), as well as transcriptional expression regulation [1, 11, 15, 17, 46].

The current research examines differential adaptation of the two forms of *T. urticae* to host plants and potential mechanisms involved. We investigated the performance of the two forms on four different crops (common bean, cotton, cucumber and eggplant). While the genome of the green form of *T. urticae* has been previously reported [17], we here also completed a genome assembly of the red form, and examined transcriptomic responses of the two forms to different plant hosts. Findings are interpreted in terms of likely contributions of different mechanisms to divergence in host plant performance between the forms.

## Results

### Different responses between the red and green forms of *T. urticae* to plant hosts

**Fig. 1:**
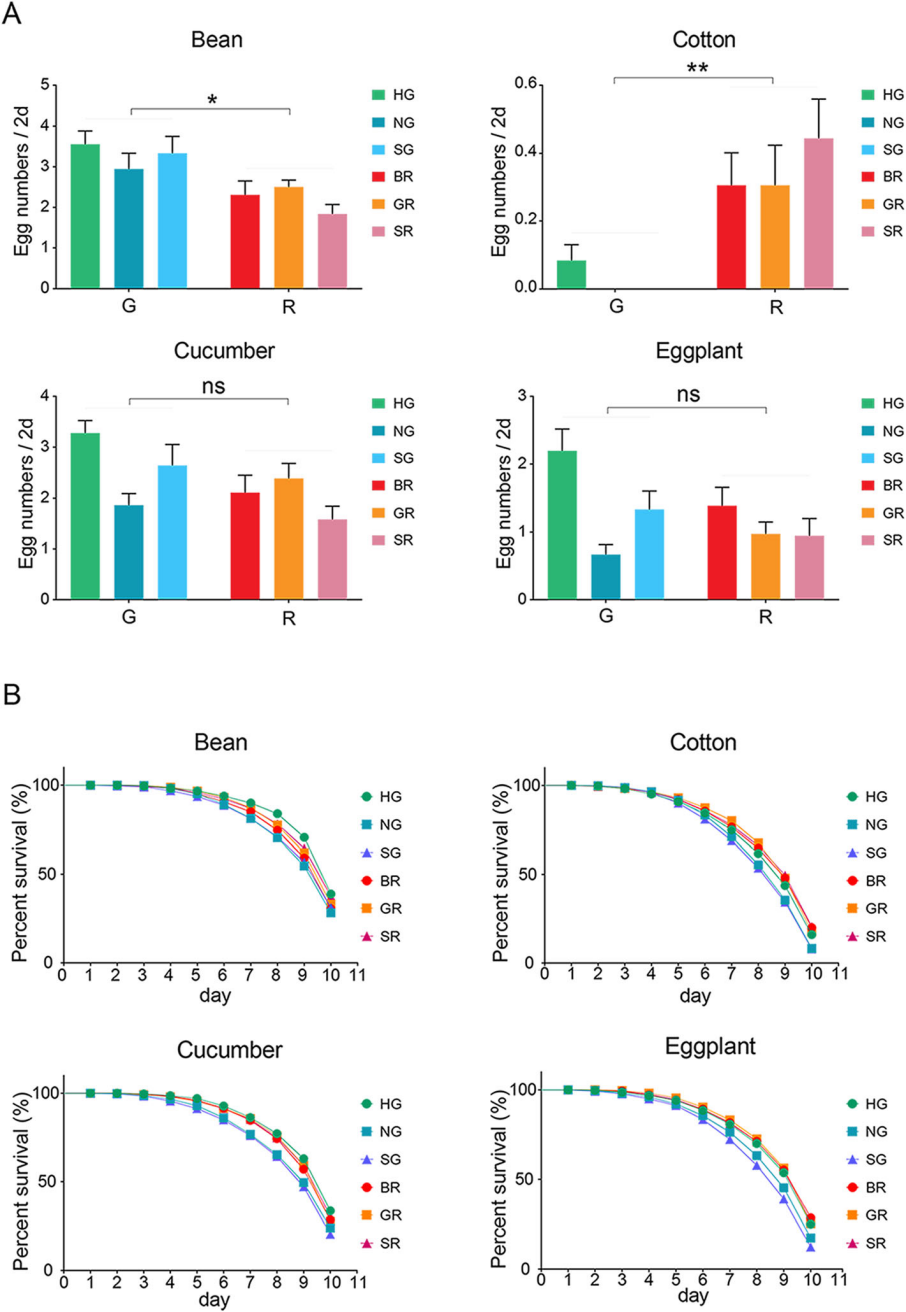
Performance comparisons between two forms of *T. urticae* feeding on the original host and three hosts to which mites were transferred. (**A**) shows fecundity comparisons of adult females of six populations in the green and red form of *T. urticae* on the original host (common bean) and each of the three other novel hosts (cotton, cucumber and eggplant) for 2 days. Results shown are mean ± SEM and statistical differences between forms. Differences between forms are based on nested analyses are indicated (*P < 0.05; **P < 0.01; ***P < 0.001), “ns” indicates not significant. G: the green form of *T. urticae*; R: the red form of *T. urticae*. (**B**) Survival comparisons (%) of 0-day-old adult females in six populations of both forms of *T. urticae* after feeding on each of the four different hosts across 10 days

Fecundity and survival were compared between the two forms. Overall, the fecundity of 2-day-old adult females of the green form of *T. urticae* was significantly higher than that of the red form after feeding on bean (Fig. 1A, Table S1; *F*_1,4_ = 15.953, *p* = 0.016), while it was significantly lower compared to the red form after feeding on cotton (Fig. 1A, Table S1; *F*_1,4_ = 36.029, *p* = 0.004). There were no significant differences in fecundity between the two forms of *T. urticae* for the other two hosts (Fig. 1A, Table S1; all *p* > 0.05). Survival of 60 newly emerged adult virgin females monitored for 10 days revealed significant differences among the two forms after feeding on cotton (Fig. 1B, Table S2). For the other hosts, differences among populations nested within forms were found (Table S2). Survival curves highlighted that one green population (HG) represented an outlier, contributing to significant differences among the three green populations of *T. urticae* when fed on each of the four host plants (Fig. 1B, Table S2; Log-rank (Mantel-Cox) tests, all *p* < 0.05). These performance differences between the two forms and among populations of the same form suggest a history of past selection on host use.

**Fig. 2:**
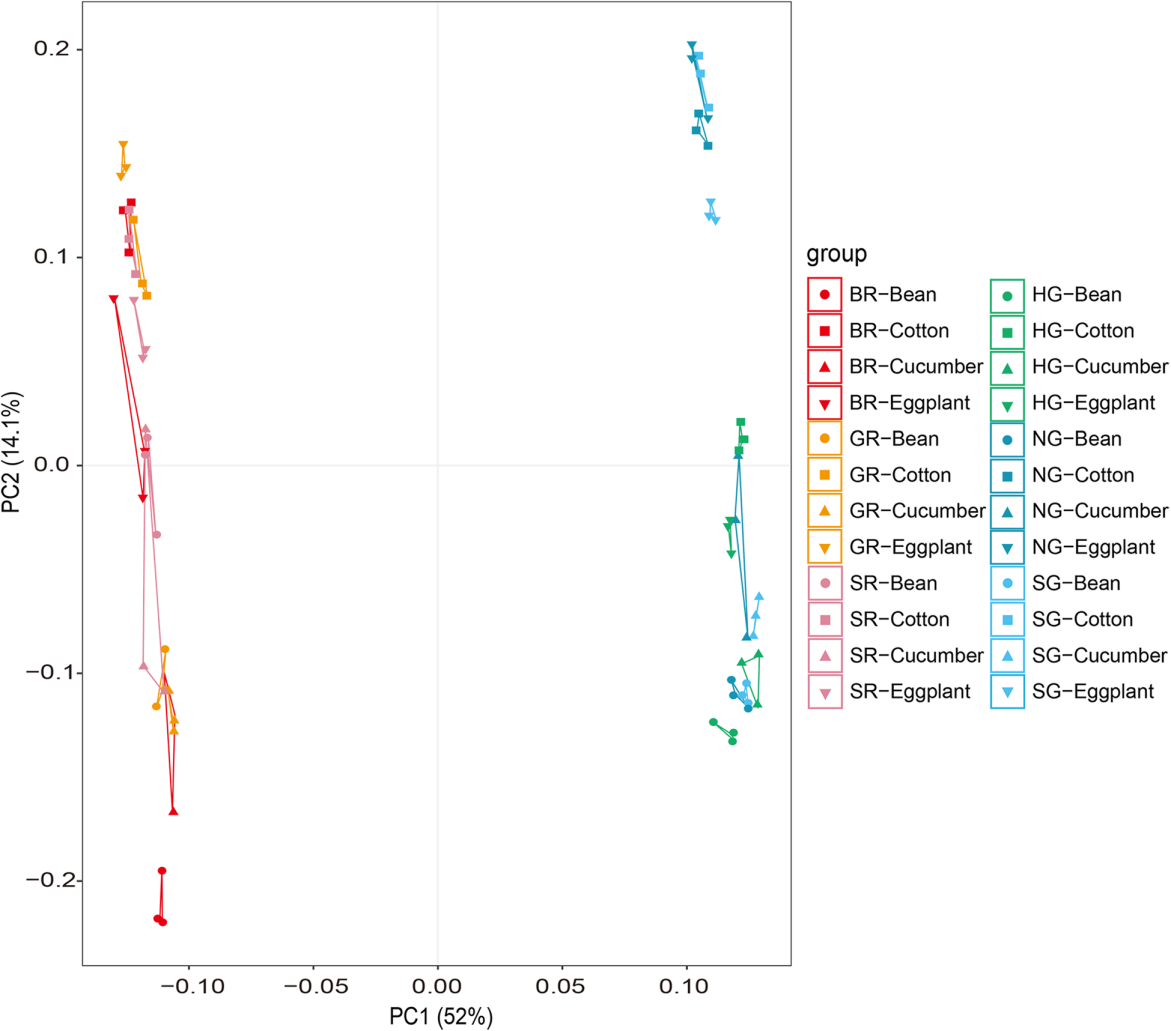
Principal component analysis among the red and green *T. urticae* populations feeding on the four hosts. PCA plot of the transcript levels from the six red and green *T. urticae* populations on the original host and three transferred hosts (cotton, cucumber and eggplant). Average FPKM values of each population fed on each of four hosts were used as input for analysis. HG (Inner Mongolia population of the green *T. urticae*); NG (Jiangsu population of the green *T. urticae*); SG (Shandong population of the green *T. urticae*); BR (Beijing population of the red *T. urticae*); GR (Guizhou population of the red *T. urticae*); SR (Shandong population of the red *T. urticae*)

Comparisons then focused on whole transcriptional responses of six populations after transfers from bean to three other hosts*.* A PCA (principal component analysis) plot showed that 52.0 and 14.1% of the total gene expression variation across six different host plant populations could be explained by the first two principal components (Fig. 2), and PC1 clearly separated these samples into two groups corresponding to green and red forms (Fig. 2), indicating overall expression patterns were substantially affected by forms when compared to hosts. Similar findings were revealed in a correlation heatmap of these genes and in hierarchical clustering analyses (Fig. S1 and S2). Moreover, the hosts of each color form cluster tended to cluster into two for the different geographic populations; bean and cucumber formed one cluster, while eggplant and cotton formed the other cluster (Fig. 2, Fig. S2).

### Comparative genomics

**Fig. 3:**
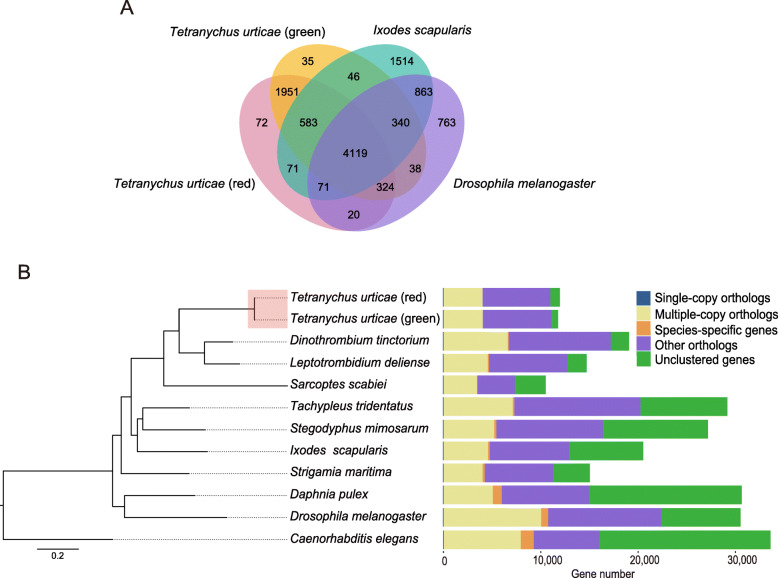
Comparative genomic and phylogenetic analyses. (**A**) Venn diagram of homologous gene families among the red form of *T. urticae*, the green form of *T. urticae*, *Ixodes scapularis* and *Drosophila melanogaster*. (**B**) Phylogenetic relationships of both forms of *T. urticae* to other species based on single-copy orthologous genes derived from full genomes. Twelve organisms were used for the analyses, including both forms of *T. urticae*, *Stegodyphus mimosarum*, *I. scapularis*, *Tachypleus tridentatus*, *Sarcoptes scabiei*, *Dinothrombium tinctorium*, *Leptotrombidium deliense*, *Strigamia maritima*, *D. melanogaster*, *Daphnia pulex* and *Caenorhabditis elegans*. The maximum-likelihood phylogenetic tree was reconstructed with 1000 ultrafast bootstraps and selected substitution models of the options “-mset” (WAG and LG). The focus (two forms of *T. urticae*) in our study are marked with red

To explore molecular differences between the forms, we first consider the whole genome of the red form of *T. urticae* based on Illumina and PacBio sequencing platforms (Table S3, Supplementary methods). Genome size was estimated with GenomeScope to range from 88 to 90 Mb, and heterozygosity varied from 0.0304 to 0.0382% (Fig. S3). The final assembly generated 90.1 Mb of reference genome that was composed of 144 scaffolds with an N50 of 12.78 Mb, and a GC content of 32.3% (Table 1). Characterization of repetitive elements, non-coding RNA prediction, and summary information of gene and family in species analyses covered here can be found in the Supplementary information (Tables S4, S5 and S6). The red form was predicted to contain 11,917 protein-coding genes (Table 1). Comparative genomic analysis revealed a high percent of orthologous gene families (96.8%) between the two forms of *T. urticae* compared to that of either the tick *Ixodes scapularis* or the fly *Drosophila melanogaster* (Fig. 3A). In addition, phylogenetic inference revealed a close evolutionary relationship between the two forms of *T. urticae* (Fig. 3B), while the tick *I. scapularis* and spider *S. mimosarum* clustered together as a sister group of Acariformes (Fig. 3B). This is consistent with the previous view that ticks and spider mites do not constitute a monophyletic group and do not have a close relationship [47]. The difference in predicted protein coding genes between the two forms will partly reflect the two different approaches used in assembly and annotation which lead to differences in data filtering. We also annotated fewer protein coding genes in the red form than the green form which relates to the different searching software used.

**Table 1:** Assembly and annotation summary statistics of the red form of *T. urticae*

	*T. urticae* (red)
Total scaffold number	144
Total scaffold length (Mb)	90.11
Scaffold N50 length (Mb)	12.78
Scaffold N90 length (Mb)	3.82
Longest length of scaffold (Mb)	22.75
GC (%)	32.33
Assembly BUSCO (%)	
C	92.2
S	87.1
D	5.1
F	1.2
M	6.5
Mapping rate (%)	
RNA-seq data	96.61
Illumina data	96.95
PacBio data	92.76
Gene annotations (number/length)	
Genes	11,917
Gene mean length (bp)	3891.9
CDSs	11,917
CDS mean length (bp)	1487.2
Exons	47,355
Exon mean length (bp)	600.1
Introns	31,730
Intron mean length (bp)	565.67

Expansion and contraction of gene families were identified in the red form of *T. urticae* genome using CAFÉ. There were 801 genes gained in 546 expanded families and 1000 genes lost in 873 contracted families in the red form (Table S7). Comparison of expanded and contracted gene families revealed that there were a few rapidly evolving families in the genomes of both the red (17: 11 expansions and 6 contractions) and green (19: 13 expansions and 6 contractions) forms (Table S7). Among all species compared here, 11 rapidly expanded families identified in the red form were mainly related to reverse transcriptase (RNA-dependent DNA polymerase), transcription factor TFIID (or TATA-binding protein, TBP), meiosis regulator and mRNA stability factor 1, and ATP-dependent DNA helicase (Table S8). Among the expanded families, 23 genes were involved in the transcription factor TFIID, which has a critical regulatory effect on eukaryotic gene expression [48].

To further obtain insight into potential linkages between gene families and biological attributes of the forms, we closely examined gene families associated with salivary catabolic enzyme, gut digestion and detoxification, which are critical for herbivory. We manually searched genes involved in saliva, digestion and detoxification in the red form’s genome based on orthologous alignment against the green form. A total of 82 salivary proteins, 82 genes encoding cysteine peptidase, 84 CYP450 genes, 103 ABC genes, 28 GST genes, and 73 CCE genes were identified in the red form (Table 2).

**Table 2:** Gene families involved in saliva, digestion and detoxification in the red and green forms of *T. urticae* genomes

	Gene family	*T. urticae* (red)	*T. urticae* (green)
Saliva		82	92^a^
Digestion	Cysteine peptidase	82	87^b^
Detoxification	CYP450s	84	81^b^
	ABCs	103	103^b^
	GSTs	28	32^b^
	CCEs	73	71^b^

^a^ The data was obtained from Huang et al. 2018 [49]

^b^ Numbers were derived from Grbić et al. 2011 [17]

### Comparing gene expression of the two forms after transfer to different hosts

**Fig. 4:**
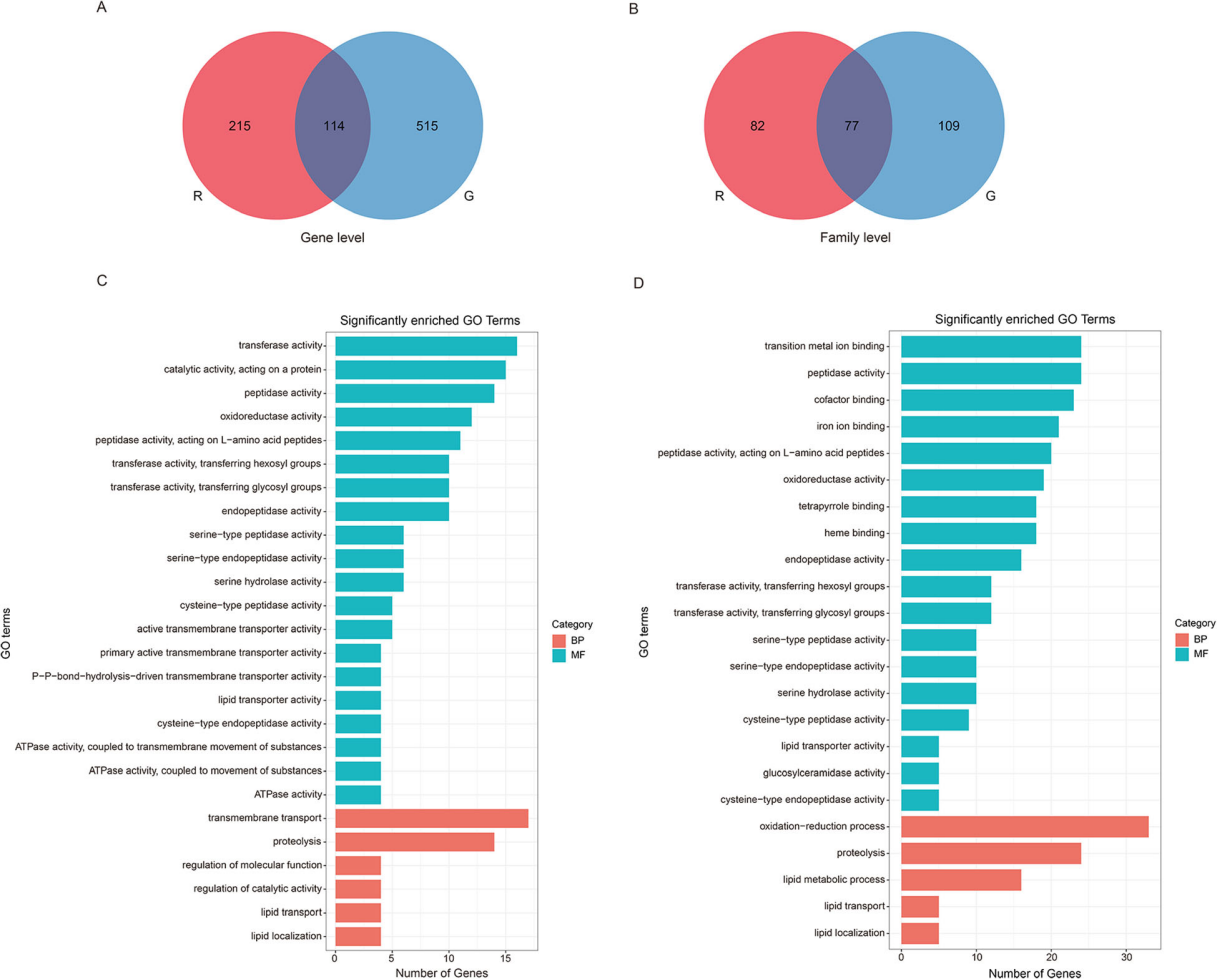
Venn-diagram showing the overlaps of DEG sets (|log2 fold change| > 1 and q-value < 0.05) between all the red and green *T. urticae* populations feeding on different transferred hosts, and GO enrichment of form-specific genes in the red or green *T. urticae* fed on the three transferred hosts. (**A**) and (**B**) are Venn diagram comparisons of combined DEGs derived from the three red and three green *T. urticae* populations fed on three hosts at gene (**A**) and family (**B**) levels. (**C**) and (**D**) indicate significant GO enrichment of form-specific DEGs from top10 GO terms with the smallest q value in each of three categories (CC, MF and BP) in the red and green *T. urticae*, respectively. The X axis represents the number of genes in each GO term, and the Y axis represents significantly enriched GO terms

To investigate the transcriptomic differences between two forms, we obtained combined DEGs (differentially expressed genes) shared by three different populations of each form when transferred to the three different hosts. Most of the DEGs were unique for the red or green form at both the gene level (65.3 and 81.9% DEGs unique for the red and green forms, respectively; Fig. 4A) and family level (51.6 and 58.6% families unique for the red and green forms, respectively; Fig. 4B), and the green *T. urticae* recruited many more DEGs or families in response to host transfers in contrast to the red form (Fig. 4A and B). Functional analyses further revealed that more than half the significant GO terms of form-specific DEGs were mainly related to catalytic activity, such as cysteine peptidase, serine peptidase, peptidase activity, transferase activity and endopeptidase activity (Fig. 4C and D, Table S9). Importantly, there were additional molecular functions and biological processes unique to the green form, including iron ion binding, heme binding, tetrapyrrole binding, transition metal ion binding, cofactor binding, lipid metabolic process and oxidation-reduction process (Fig. 4D, Table S9). The red form possessed some specific GO terms involved in transporter activity (Fig. 4C, Table S9). These findings suggest different biochemical responses in the two forms following host transfers.

**Fig. 5:**
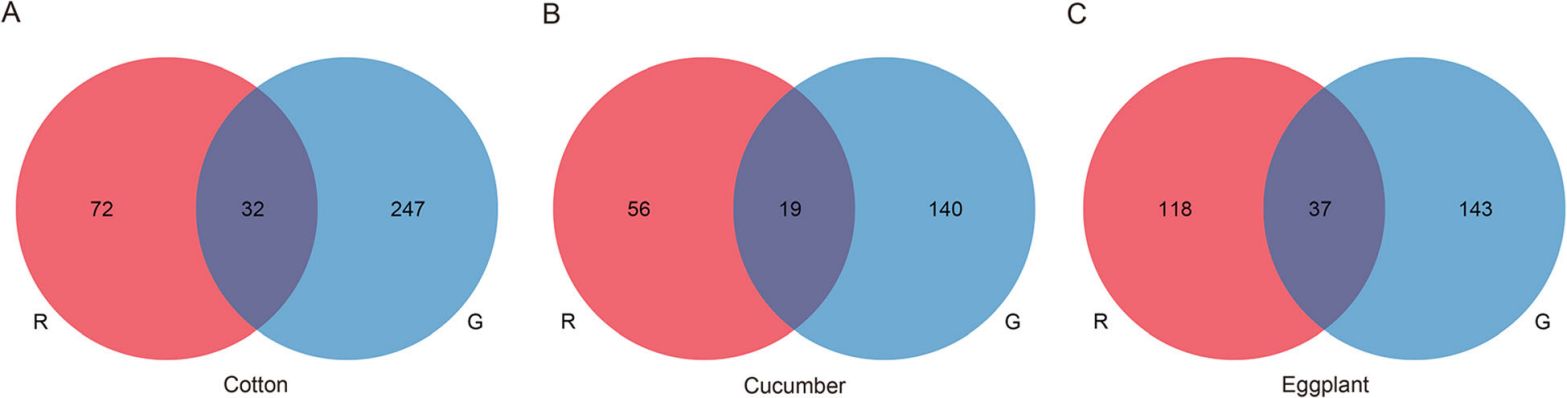
Venn diagrams of DEGs between the red and green *T. urticae* feeding on the same transferred host. The DEG sets of the red or green *T. urticae* on each of three transferred hosts: (**A**) cotton, (**B**) cucumber and (**C**) eggplant were derived from overlaps of their three populations. The red *T. urticae* is indicated in red and the other color represents the green *T. urticae*

We then compared transcriptomic expression differences between the two forms after transferring from bean to cotton, cucumber or eggplant. The majority of DEGs were different between the two forms of *T. urticae* for each host transfer at the gene level (69.2, 74.7 and 76.1% of DEGs unique for cotton, cucumber and eggplant in the red form, with equivalent figures for the green form of 88.5, 88.1 and 79.4%; Fig. 5A-C). In addition, a few genes were not affected by color form, but shared by all populations of both forms, and these presented similar expression patterns of upregulation or downregulation following the same host transfer (100, 94.7 and 97.3% of overlapped DEGs following cotton, cucumber or eggplant transfers; Table S10). We also counted gene families of these DEGs grouped via OrthoFinder and found that they showed mainly form-specific changes in transcriptomic responses following host transfer (Fig. S4). These findings further highlight the different genes and functions recruited by the forms when responding to the same host transfer.

### Comparing gene expression on hosts within the two forms

**Fig. 6:**
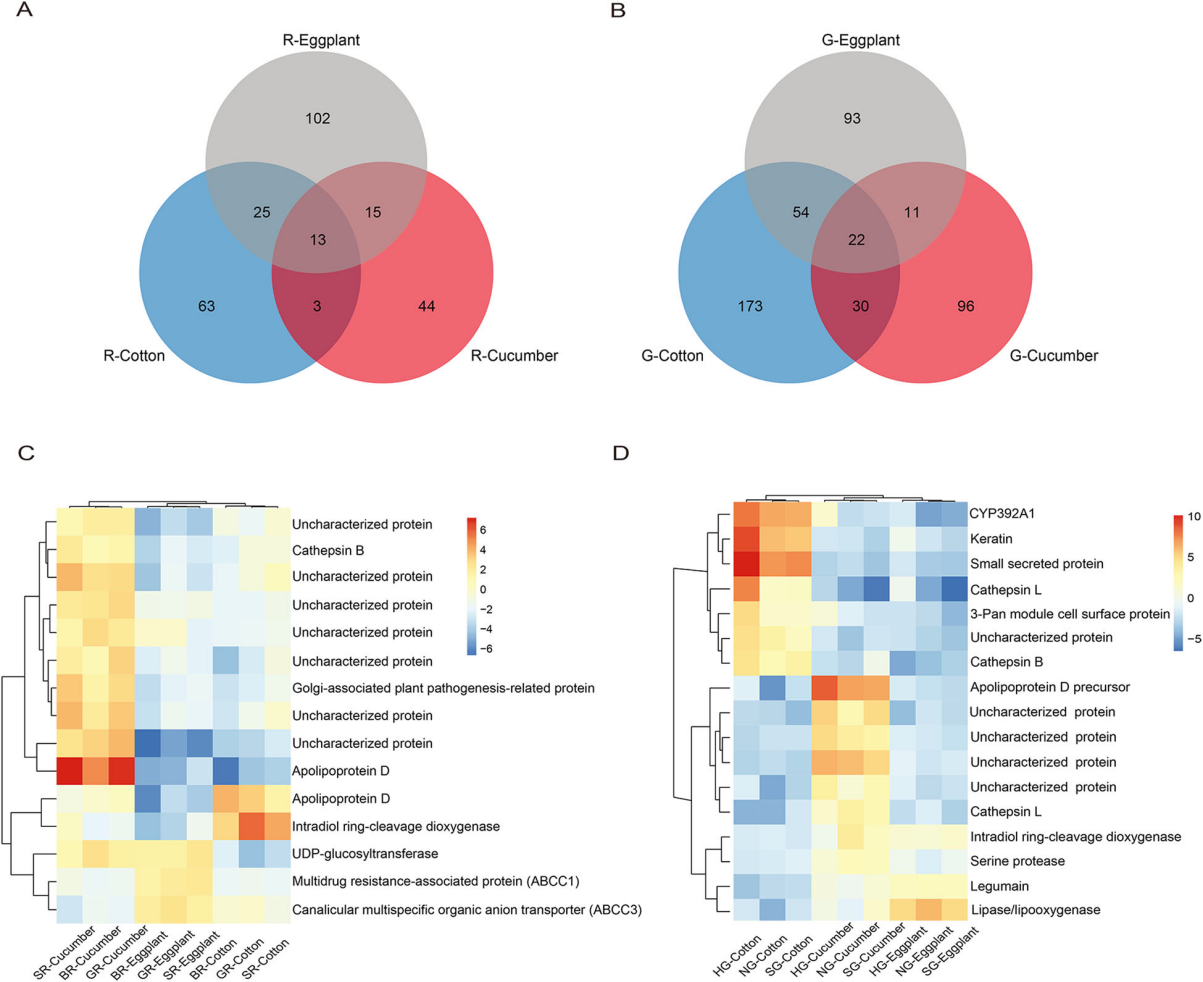
Venn diagrams of DEGs in the red or green *T. urticae* feeding on different transferred hosts, and heatmap of DEGs with constitutively different expression for the red and green *T. urticae*. (**A**) and (**B**) represent the DEG overlaps for the red or green *T. urticae* at the gene level across the three transferred hosts, respectively. The data used for analyses derived from gene sets shared by the three red or green *T. urticae* populations fed on the same transferred hosts. (**C**) and (**D**) represent heatmaps of 15 and 17 DEGs with constitutively different expressions for the red or green *T. urticae*, respectively. Constitutive differences were considered as only genes significantly up-expressed or down-expressed in one transferred host compared to all others analyzed here for the red or green *T. urticae*

In order to explore how the two forms may adapt to different hosts, we investigated the differences in gene expression of either the red or green form of *T. urticae* in response to the hosts (Fig. 6). When we compared transcriptomic responses to the three host transfers in all populations of the red or green form at the gene level, most DEGs in each color form showed host specific expression (Fig. 6A and B, Table S11); a few of DEGs shared by the two and three host transfers overlapped in both forms, and the majority of these genes were detected responding in the same way to two or three hosts with consistently up- or down-regulated patterns (41 out of 265 DEGs and 89 out of 479 DEGs for the red and green *T. urticae*, respectively; Fig. S5A and B). In addition, gene families of these genes showed mainly host-specific transcriptomic responses to different transfers for the red or green form (Fig. S5C and D). Functional enrichment of these host-specific DEGs also showed that different transfers could lead to most host-dependent GO terms in the red or green *T. urticae* (Fig. S6, Table S12). These results indicated that the diverse characters of host plants played important roles in rearranging transcriptomic changes of the red or green *T. urticae*.

We found that a small number of DEGs showed constitutive differences in expression when considering only genes significantly up-regulated or down-regulated on one host after transfer (Fig. 6C and D). There were 15 and 17 DEGs constitutively expressed for the red and green forms, respectively, mainly up-regulated after feeding on cucumber but down-regulated after feeding on cotton or eggplant (Fig. 6C and D). Many of them were related to detoxification and digestion, such as ABC transporter, cathepsin B and intradiol ring-cleavage dioxygenase for the red form, and cytochrome P450 monooxygenases, cathepsin L, cathepsin B, intradiol ring-cleavage dioxygenase and serine protease for the green form of *T. urticae* (Fig. 6C and D, Table S13). In addition, three of the 15 and 17 DEGs showing constitutive differences in expressions among the three transfers across the red and green *T. urticae* populations respectively were used for quantitative validation of transcript levels via qRT–PCR (quantitative reverse transcriptase-polymerase chain reaction) (Fig. S7). These six genes showed significant up-regulation following cucumber transfer but down-regulation following cotton and eggplant for both forms (Fig. S7), consistent with the transcriptome sequencing data (Table S14).

To gain additional insights into host-specific transcriptomic patterns resulting from different transfers, we performed k-mean clustering analyses of transcriptomic responses of three populations of each color form following the three host transfers. Four clusters were evident for the red color form (Fig. S8, Table S15). Two main patterns of changes in expression were evident for the red *T. urticae* (Fig. S8). Overall, 197 out of the 265 DEGs analyzed in cluster 1 reflected significant down-regulation after feeding on cotton and cucumber, as well as up- and down-regulation when feeding on eggplant; cluster 2 to 4 consisted of 19, 42 and 7 DEGs exhibited specific up-regulation when feeding on eggplant, cucumber and cotton, respectively (Fig. S8). For the green form, a total of 479 DEGs within six clusters showed three major transcriptomic patterns (Fig. S8, Table S15). Cluster 1 of 175 DEGs showed host-dependent down-regulation in response to the three different host transfers, while 209 DEGs in cluster 3 showed specific up-regulation when feeding on eggplant, cucumber or cotton; 8 and 15 DEGs in cluster 4 and 6, as well as most of 65 DEGs in cluster 2 exhibited specifically up-regulated after transferring from bean to cucumber, cotton or eggplant (Fig. S8). Cluster 5, with 7 DEGs, did not show host plant specificity but a common expression pattern following host transfers (Fig. S8).

### Transcriptomic patterns of the saliva, detoxification and digestive genes on the different host plants

**Fig. 7:**
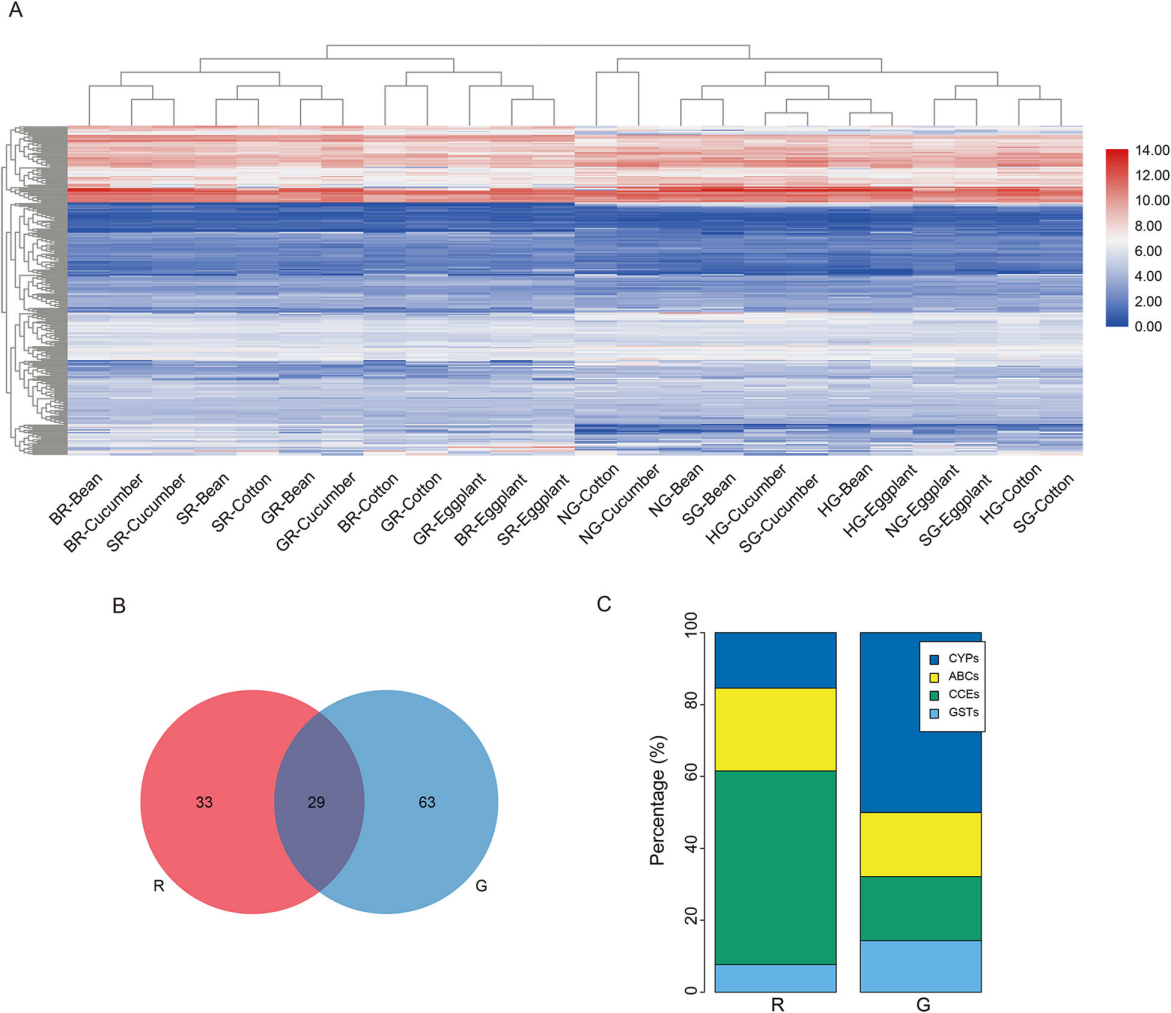
Heatmap clustering and comparisons of DEGs related to saliva, digestion and detoxification between the red and green *T. urticae* among different transfers. (**A**) Heatmap shows the mean expression level of one-to-one orthologous genes between the red and green *T. urticae* feeding on the original host and three transferred hosts for 2 days. Red represents high regulation, and blue represents low regulation. Hierarchical clustering of these DEGs was performed based on Pearson’s correlation as the distance measure and the complete cluster method. (**B**) The DEGs associated with these families between the red and green *T. urticae* across all populations and transferred hosts. The red *T. urticae* is indicated in red and the other color represents the green *T. urticae*. (**C**) Comparison of form-specific detoxification genes between two forms of *T. urticae*. R, the red form of *T. urticae*; G, the green form of *T. urticae*

To provide insight into expression profiles of genes involved in saliva, digestion and detoxification and their possible involvement in form-specific responses to hosts, clustering analysis of 248 one-to-one genes in these categories among six populations of the red and green forms was performed when presented on bean and the three other hosts (Fig. 7A). The expression pattern of these genes clustered together based on the host plant to which each color form was exposed at the lowest hierarchical level, followed by the red or green form at a higher hierarchical level (Fig. 7A). This result highlights the innate differences in gene expression between the two forms.

**Fig. 8:**
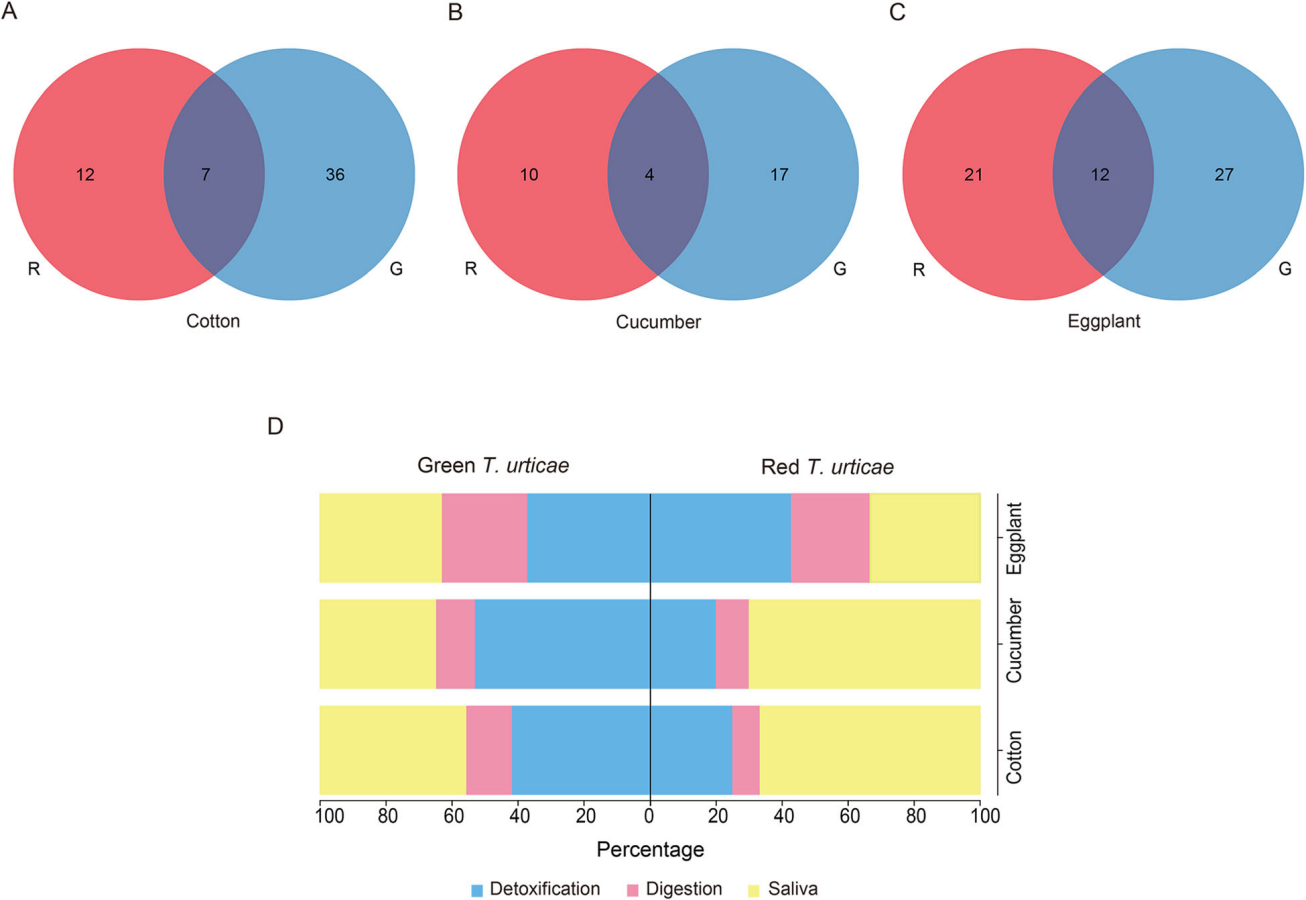
Venn diagrams and form-specific DEGs associated with saliva, digestion and detoxification between the red and green *T. urticae* feeding on the same transferred host. The DEGs of the red or green *T. urticae* on each of three transferred hosts were derived from overlaps of their three populations: (**A**) cotton, (**B**) cucumber and (**C**) eggplant. The red *T. urticae* is indicated in red and the other color represents the green *T. urticae*. (**D**) indicates different proportions of form-specific DEGs in these families for the red or green *T. urticae* on each of the three transfers

Transcriptomic expressions of the saliva, detoxification and digestive gene families between the two forms were further compared. These families showed mainly form-specific plastic transcriptomic responses to the host plants (Fig. 7B), and both forms recruited many genes of the saliva and detoxification families after transferring from bean to cotton, cucumber, or eggplant, followed by digestive genes (Fig. S9A-C, Table S16). We also found that form-specific subfamilies within detoxification functions were differently assigned to two forms after host transfer (Fig. 7C, Table S16). More than half of the specific DEGs were associated with CCEs, followed by ABCs for the red form, while the green form was associated with changes in many CYP450s and GSTs in response to different host plants (Fig. 7C, Table S16). We therefore wondered it different hosts induce different defensive responses between two forms. When we focused on the DEGs in the saliva, detoxification and digestive gene families responding to different host plants, the majority of DEGs were different between the two forms of *T. urticae* (63.2, 71.4 and 63.6% of DEGs unique to the red form for cotton, cucumber and eggplant respectively, 83.7, 81.0 and 69.2% unique to the green form respectively, Fig. 8A-C), and almost all overlapped DEGs between the two forms following the same transfers had similar expression patterns of consistent up-regulation or down-regulation (Table S17). Functional statistics of host-specific DEGs revealed that salivary proteins were mainly recruited by the red form for dealing with cotton and cucumber, while the green form recruited salivary and detoxification enzymes for these hosts (Fig. 8D, Table S17). Eggplant transfers involved both forms recruiting members of all three families (Fig. 8D, Table S17). As indicated previously, the gene families responding to different host plants were inconsistent in the red or green *T. urticae*. For the red form, 47 of the 442 (10.6%) DEGs analyzed were differentially expressed depending on host transfer, while for the green form the equivalent figures were 73 of the 456 (16%) DEGs analyzed; most of these DEGs were specific to different hosts for both forms (Fig. S9D and E). These findings further highlight the interaction between form and host.

## Discussion

### Gene expression differences between forms and hosts of spider mites

Adaptation can result in changes in the expression of genes both constitutively and plastically [50, 51]. While constitutive expression changes are independent of the environment, transcriptional plasticity represents an induced response upon exposure to a new environment, which has been suggested to substantially contribute to adaptation [52–54]. In the green form of *T. urticae,* transcriptional studies have provided new insights into effects of exposure of new hosts on the transcriptome [1, 17, 46]; research suggested that transcriptional signals of *T. urticae* were affected by switching mites to a tomato host, and that genetic adaptation to the novel host led to both plastic and constitutive transcriptional changes [1].

Our study highlighted transcriptional changes of the red and green form of *T. urticae* feeding on three hosts. We found evidence of both inherent transcriptional differences between the red and green forms of *T. urticae* and form-dependent plastic transcription expressions at both gene and family levels, as well as their functional enrichments (Fig. 4). We also discovered that the majority of DEGs in response to the different host plants were not shared in the red or green form of *T. urticae* at both gene and family levels (Fig. 6A and B, Fig. S5), highlighting the fact that the two forms presented specific responses to host transfers. Although we also observed a small number of genes showing conservative expression changes among different transfers within each form of *T. urticae* (Fig. 6C and D), our results suggest that transcriptional plasticity might play a role in adaptation to new host environments and contribute to the polyphagous nature of the two-spotted spider mite.

Plasticity of gene expression has previously been linked to the evolution of polyphagy in arthropod herbivores [1, 31, 46, 55]. Previous results have shown transcriptomic plasticity in the green form of *T. urticae* following long-term acclimation to host switches [46], where host specific responses occurred when the green form was transferred from its original host of common bean to lima bean, soybean, cotton, tomato or maize. Plasticity in gene expression has also been linked to the ability of larvae of the comma butterfly to feed on a divergent host [31]. Plastic changes in gene expression most likely benefit herbivores in surviving on novel hosts when they are first exposed to them [53, 56]. Eventually plastic changes increasing fitness on a novel host may develop to become genetically determined environmental responses [57] or become constitutively expressed under selection [58]. A study on the transcriptional changes of the green form of *T. urticae* when exposed to tomato found substantial genetic variation in constitutive expression compared to plastic variation in transcript regulation [1].

We found some evidence that forms differed for traits including fecundity and survival when present on two hosts (Fig. 1). However, we also found evidence of variation among populations with the two forms, highlighting the fact that differences among populations exist in host fitness that are independent of the spider mite form. One challenge in these experiments is that we were unable to access populations of the two forms from the same geographic location except in one case. This meant that form differences were confounded by geographic location and selection for different patterns of host use within the two forms. It will be interesting to follow long-term transcriptional adaptation of both the red and green forms when the mites are exposed for multiple generations to different hosts, and to monitor changes in the ability of the mite populations to suppress or attenuate plant defenses. Parallel experiments in the red and green forms would be of particular interest because of the different transcriptional profiles that occur in these two forms, suggesting strong effects of genetic backgrounds represented by the two forms as evident from our genomic sequencing work.

### Effect of host exposure of *T. urticae* forms on specific genes/ families

Arthropod herbivores can develop a set of defensive system to cope with toxic effects of host plants, of which the detoxification and transport families are best studied [9, 10]. Previous molecular studies showed that adaptation by polyphagous herbivores following host plant transfer was closely related to the differential expression of genes coding for metabolism, conjugation and translocation in the detoxification process [31, 59, 60], and transcriptional expression of these genes could be modified largely by herbivores after feeding on different host plants with varying nutritional and toxic compounds [31, 60]. In spider mites, preoral digestion and intracellular breakdown are considered important when mites take nutrition from host plants, and we paid attention to how genes in families related to saliva, digestion and detoxification responded to host transfers that presented form- and host-specific transcription changes as described in overall expression patterns (Fig. 7C, Fig. S9D and E, Table S11).

We found that forms and host transfers affected the expression of genes of these families (Figs. 7C and 8D), suggesting that plastic changes in host induced expression may be related to host characteristics such as defensive chemicals. Previous studies have indicated that different plant families could produce various toxic metabolites and anti-nutritional chemicals in their defenses against herbivores [2, 61]. Adaptation to different host plants with known defense compounds is thought to involve changes in transcriptomic expression patterns of specific genes [46]. For instance, gossypol is a well-characterized phytoanticipin in cotton, and both UDP-glycosyltransferases (UGTs) and P450-oxygenation may play important roles in gossypol detoxification [62, 63]. In our study, 3 UGTs (*tetur08g03000* (teturUGT42), *tetur04g02350* (teturUGT13) and *tetur32g01250* (teturUGT75)) and 3 CYP genes (*tetur07g06460* (*CYP392A3*), *tetur47g00090* (*CYP392A9*) and *tetur08g06170* (*CYP387A1*)) were significantly up-regulated when the green form of *T. urticae* fed on cotton (Table S12). It has been reported that the CYP392 family belonging to the CYP2 clan responds strongly to host plant changes and is also associated with resistance to acaricides of the green form of *T. urticae* [16]. Interestingly, one *CYP392A3* ortholog in the red *T. urticae* was significantly up-regulated to cotton after transfer from the original host (Table S11 and S17), and we also found that two other genes of this family were significantly up-regulated in the red and green *T. urticae* following transfer to eggplant (Table S11). Considering that cotton and eggplant are poor hosts for both forms of *T. urticae* when compared to common bean and cucumber (Fig. 1), the significant up-regulation of these CYP392 family genes suggests that this family may help spider mites cope with poor hosts.

In addition to classical detoxification and digestive genes which have been implicated in transcriptional response following host plant transfers [1, 11, 15, 46, 55], some additional genes thought to be introduced to the arthropod genome by horizontal gene transfer were also identified in our study. The enzymatic repertoires of phytophagous arthropods can be expanded by genes horizontally transferred from microbial species, which can promote adaptation to novel host plants likely through changes in phytotoxin processing [18]. UGT genes, coding for important conjugation enzymes in many organisms, have been acquired via horizontal gene transfer from bacteria to the green form of *T. urticae* genome [64]. We discovered that a total of 8 UGTs showed host-specific up-regulation in the red *T. urticae*. Moreover, intradiol ring-cleaving dioxygenases represent a novel gene family acquired via horizontal gene transfer into spider mites [17, 60]. Several studies have revealed that this unique gene family can exhibit a strong transcriptional response to various host plants and acaricides [17, 19, 60, 65]. Notably, we found 2 DEGs (*tetur19g02300*, *tetur19g03360*) coding for intradiol ring-cleaving dioxygenases that showed significant cotton-specific up-regulation in the three green *T. urticae* populations (Table S11). Furthermore, we found 2 DEGs (*tetur01g15760*, *tetur01g06600*) coding for the major facilitator superfamily specifically up-regulated following cotton and eggplant transfer in the green form of *T. urticae* (Table S11). These genes were previously not known to be involved in arthropod detoxification but now appear to be differentially expressed following herbivore transfer [1, 17, 60, 64, 66], which further supports the view that adaptive evolution in phytophagous spider mites may be driven at least partly by horizontal gene transfer [17, 60, 64, 67]. Transcriptional changes of these lesser-studied gene families have also been noted in other studies [1, 46, 60]. Combined with our data, these findings strongly suggest that the two-spotted spider mites use laterally acquired genes in their detoxification system to overcome plant defenses.

## Conclusions

In our study, we found substantial transcriptional differences between the two forms of *T. urticae* regardless of their geographical origin. We established some links between the performances of the two forms after transfer to different host plants, although there was also variation among populations within the same form. In order to examine the transcriptional responses in more detail, we reported a completely assembled 90.1-Mb genome of the red form of *T. urticae*, and annotated major saliva, digestion and detoxification genes. Gene or family comparisons for transcriptional data showed differences between the forms that likely contribute to performance on the different diets, and transcriptional plasticity appears to be used by both forms of *T. urticae* to respond to a set of host plants even though the sets of genes involved are mostly different*.* Together, these findings contribute to an understanding of adaptation of mites to novel hosts and they highlight the importance of considering intraspecific variation as represented by the two forms of *T. urticae*.

## Methods

### Mite strains and host plant

The red form of *T. urticae* used for genome sequencing was originally collected from dryland willow (*Salix matsudana f. tortuosa*) leaves in Kunming, Yunnan province of southwest China (25°12′ N, 102°75′ E) in 2012, and maintained on leaves of common bean plants (*Phaseolus vulgaris*) in the laboratory for up to 4 years before sequencing. A combination of morphology and molecular data was used to identify the spider mite based on the morphological characteristics of the aedeagus of the male mite, as well as sequences of the ribosomal gene ITS and the mitochondrial gene COI. Morphological identification from a glass slide of the male mite was performed by the Japanese expert Professor Tetsuo Gotoh (Ryutsu Keizai University) in 2015; the molecular identifications were carried out by using primers COI-F (5′- AAGAGGAGGAGGAGACCCAATT − 3′) and COI-R (5′- AAACCTCTAAAAATAGCGAATACAGC − 3′) for mitochondrial gene COI, and primers rDO2 (5′- GTCGTAACAAGGTTTCCGTAGG − 3′) and HC2 (5′- ATATGCTTAAGTTCAGCGGG − 3′) for ribosomal ITS. To distinguish the contribution of geographic populations versus forms, a total of 6 populations of the red and green forms of *T. urticae* were used for performance tests and transcriptional studies, including 3 wild-collected red forms derived from peanut (*Arachis hypogaea* L.) leaves in Shandong (36°24′ N, 117°11′ E) (SR), tomato (*Solanum lycopersicum* L.) leaves in Guizhou (26°42′ N, 106°66′ E) (GR) and eggplant (*Solanum melongena* L.) leaves in Beijing (39°91′ N, 116°66′ E) (BR), as well as 3 green forms originally collected from peanut (*Arachis hypogaea* L.) leaves in Shandong (36°24′ N, 117°11′ E) (SG), pumpkin (*Cucurbita moschata* D.) leaves in Inner Mongolia (40°79′ N, 111°79′ E) (HG) and soybean (*Glycine max* L.) leaves in Jiangsu (32°03′ N, 118°84′ E) (NG) provinces in China (2015–2020), and these were also maintained on bean hosts. Several hundred spider mites contributed to populations of each form, and these were maintained in culture at a size of several thousand. Populations used in experiments were reared at 25 ± 1 °C with 60–70% relative humidity (RH) and a 16:8-h (light/dark) photoperiod in a laboratory.

### Performance test

To monitor responses to the different hosts, fecundity and survival tests were carried out with both forms of *T. urticae* feeding on the common bean and three other plants to which they were transferred. The forms of *T. urticae* that had been maintained on common bean (original host) (*Phaseolus vulgaris* L. cultivar Sucaidou 11) were tested on this host compared to cotton (*Gossypium hirsutum* L. cultivar Nannong 10), cucumber (*Cucumis sativus* L. cultivar Lufeng) or eggplant (*Solanum melongena* L. cultivar Suquqi). These plant species constitute economically important but unrelated plants fed on by spider mites. To generate mites, approximately 150 mated adult females were placed on a leaf disk of common bean and allowed to oviposit for 3 h. After that, females were removed and their eggs were left to develop to become adult virgin females used for further experiments (male offspring were removed). We focused on fecundity in these mites over periods of 2 days, and survival over a longer period.

For fecundity measurement, 36 newly emerged adult virgin females of each form of *T. urticae* reared on original bean were each placed on a separated leaf disks (with diameter 15 mm) in petri dishes (with diameter 120 mm) for each of the above four host plants. Egg numbers per mite were computed after 2 days of oviposition. For survival tests, 60 newly emerged adult virgin females of each form of *T. urticae* were placed on a whole leaf (approximately 7 cm in diameter) of a host in a petri dish (diameter 90 mm), and survival of adults was monitored for 10 days from the date of transfer. Petri dishes were held at 25 ± 1 °C with 60% RH and a 16:8-h (light/dark) photoperiod.

We ran Generalized Linear Models (GLM) to examine differences of fecundity and survival among the two forms on the same host, in which populations were treated as a nested random factor within color form. The analyses were undertaken in IBM Statistics SPSS 25.0. A log rank (Mantel-Cox) test was used to assess differences in survival among different populations within the green form. The figures were made with GraphPad Prism v7.02 (San Diego, California USA).

### Genome sequencing and assembly

Approximately two thousand adult females derived from a red strain of *T. urticae* which had been inbred by sib mating for 10 generations were used for genome sequencing. High-quality genomic DNA was used to build Illumina and PacBio libraries, and subsequently these libraries were sequenced on Illumina HiSeq X Ten and PacBio Sequel platforms (Table S3). Genome size of the red form of *T. urticae* was estimated by *k*-mer analysis with GenomeScope v1.0.0 [68]. The genome assembly was performed using Flye v2.4.2 and Canu v1.3 [69, 70]. Genome completeness was assessed based on Benchmarking Universal Single-Copy Orthologs (BUSCO) [71] analyses against the arthropod dataset (*n* = 1066), and Illumina data, PacBio long reads as well as RNA-seq data (data derived from our previous research, 10.1007/s10493-017-0188-9). The details on genome sequencing and assembly are given in the Supplementary methods.

### Protein coding gene annotation

For homology-based prediction, Diamond v0.9.18 [72] against the UniProtKB (SwissProt and TrEMBL) database with e-value of 1e^− 5^ (−sensitive -e 1e–5) was used to improve gene models that were obtained from the protein sets of 7 species (*Drosophila melanogaster* (Insecta: Diptera), *Ixodes scapularis* (Arachnida: Acari), *Tetranychus urticae* (green form, Arachnida: Acari), *Sarcoptes scabiei* (Arachnida: Acari), *Stegodyphus mimosarum* (Arachnida: Araneae), *Daphnia pulex* (Branchiopoda: Cladocera), and *Strigamia maritima* (Chilopoda: Geophilomorpha)). For ab initio prediction, Augustus v3.3 [73] and GeneMark-ET v4.33 [74] were trained using BRAKER v2.1.0 [75] with RNA-seq data. For transcriptome-based evidence, previously assembled genome-guided RNA data were used in annotation of gene sets. Finally, protein-coding genes were identified by integrating homology-based, ab initio, and transcriptome-based prediction with the MAKER v2.31.10 annotation pipeline [76]. Protein domains were also identified with InterProScan 5.30–69.0 [77] by searching the Pfam [78], Gene3D [79], Superfamily [80], and CDD [81] databases.

### Gene family evolution analyses

We searched orthogroups using OrthoFinder v2.2.7 (−f proteins -t 16 -a 16 -S diamond) [82]. Single-copy gene families, aligned using MAFFT v7.394 [83] with the L-INS-I method, and trimmed using trimAl v1.4.1 [84], were used to perform the phylogenetic analyses. A maximum-likelihood phylogenetic tree was reconstructed via IQ-TREE v1.6.3 [85] with 1000 ultrafast bootstraps (UFBoot) [86] and 1000 SH-aLRT replicates [87] estimated. A set of protein substitution models with the options “-mset” (WAG and LG) were selected, and we used the relaxed hierarchical clustering algorithm [88] with -m MFP + MERGE -mset LG -rcluster 10. We also estimated species trees using ASTRAL v5.6.1 [89] based on gene trees. CAFÉ v4.2 [90] was used to analyze expansion and contraction of gene families in the red form of the *T. urticae* genome, and birth and death rates were calculated based on the lambda parameter.

### Functional family annotation

Genes coding for saliva proteins, detoxification and digestion (cysteine peptidase) in the red form of the *T. urticae* genome were identified with MMseqs2 (Many-against-Many sequence searching) [91], according to known reference sequences derived from the green form of the *T. urticae* genome and research from Jonckheere et al. [92] and Huang et al. [49]. The preliminary annotation was performed based on a blastp search with parameters ‘-format-mode 2-alignment-mode 3-num-iteration 4-min-seq-id 0.5 -e 0.001, and the tblastn alignment was further used for manual search with parameters ‘-alignment mode 3-num-iterations 4-min-seq-id 0.5 -e 0.001. All candidates aligned in the red form of *T. urticae* genome were manually checked based on the “blast reciprocal best hit” approach to known putative gene sets in the green form of the *T. urticae* genome. Specifically, to ensure the accuracy of preliminary prediction, we identified firstly the candidates according to the best hit, and these candidates in the first round showed a high cut-off of at least 90% identity based on blastp; tblastn was then used to find the fragmented matching candidate sequences in all reference sequences followed by the first step. The selection of these segments was limited to cover the best matching regions with alignment that had at least 90% of their reference sequence aligned with a cut-off of at least 80% identity. If multiple fragment sequences with a few different bases at the overlapping site were found during manual annotation, we filled undefined gaps with the bases of the reference sequence corresponding to that position in the green form of *T. urticae*, and alignments with the same start and end positions to blastp hits in tblastn search were removed. Finally, the results from blastp and tblastn were integrated as candidate genes related to saliva proteins, detoxification and digestion (cysteine peptidase) in the red form of *T. urticae* genome.

### Transcriptomic sequencing and gene expression analyses

To gain insight into transcriptome profiles after host transfers, newly emerged adult virgin females were prepared according to the method described above, and samples for RNA-seq were prepared from 120 2-day-old adult females of both forms of *T. urticae* (six lines) feeding on the initial bean host and cotton, cucumber or eggplant hosts after they had been left on these hosts for 2 days. Three biological replicates for each host were obtained, and RNA was isolated from a pool of 100 adult females using TRIzol reagent (Invitrogen, USA) according to the manufacturer’s protocols. RNA quantity of each sample was assessed based on the RNA Nano 6000 Assay Kit of the Bioanalyzer 2100 system (Agilent Technologies, CA, USA). A total of 1 μg RNA per sample was used for sequencing library. The mRNA was purified from total RNA using poly-T oligo-attached magnetic beads, and the cDNA library was sequenced using the Illumina Novaseq platform and 150 bp paired-end reads were generated. The clean data was obtained by removing reads containing adapters, poly-N and low-quality reads of raw data.

The clean reads of the three green *T. urticae* populations (SG, HG and NG) were mapped to the reference green *T. urticae* genome (available link can be found in https://bioinformatics.psb.ugent.be/gdb/tetranychus/Tetranychus_urticae.main_genome_200909.scaffolds.fasta.gz), and the clean reads of the three red *T. urticae* populations (SR, GR and BR) were mapped to the reference red *T. urticae* genome (Yunnan population, see availability of data and materials) using Hisat2 v2.1.0 [93], and the aligned reads of each sample were assembled by StringTie v1.3.3b [94]. The reads numbers mapped to each gene were counted with featureCounts v1.5.0-p3 [95], and then quantification of transcript abundance (FPKM, Fragments Per Kilobase of transcript sequence per Millions base pairs) of each gene was undertaken based on the length of the gene and reads count mapped to this gene. Differential expression analyses were performed by comparing populations of three bean replicates to three replicates of the other host for each form using the DESeq2 v1.16.1 [96], and DEGs were identified with a cutoff of 0.05 for Benjamini-Hochberg adjusted P -values and absolute log2 fold changes (FC) at 1 [97]. GO enrichment analysis of the DEGs were implemented by the clusterProfiler v 3.4.4 in R package, and significant enrichment was defined with an corrected *P*-value ≤0.05. One to one orthologs between two forms were identified using all-to-all best reciprocal blastp hits with e-value cutoff of 1e-5 based on NCBI (National Center for Biotechnology Information) BLAST+ program (ftp://ftp.ncbi.nlm.nih.gov/blast/executables/blast+/LATEST/). A PCA plot was conducted using these one-to-one genes with the prcomp function in R v3.4.1 (R Core Team 2017) [98], and the dendrogram cluster and correlation heatmap were also performed with the hclust and corrplot functions in R, respectively.

### Hierarchical clustering analyses

Clustering heatmap was applied to explore the responses of both forms of *T. urticae* to different transferring hosts based on the pheatmap package within R. The cluster number used for the k-means clustering was approximately estimated based on the elbow method (method = “wss”, k value ranging from 1 to 10) [99]. The relative transcription levels of differentially expressed genes among three transferring hosts compared to common bean in the three red or green populations of *T. urticae* were used as input for k-means clustering. Hierarchical clustering analyses of genes involved in saliva, digestion and detoxification were performed with Pearson’s dissimilarity for distance measure and complete linkage method using software TBtools v0.6732 [100]; the genes with no expression were discarded.

### Assays of quantitative PCR

Total RNA with three biological replicates for each red or green population of *T. urticae* fed on four hosts was isolated using TRIzol (Invitrogen), and cDNA was synthesized using PrimeScriptTMRT reagent Kit with gDNA Eraser (TaKaRa). Quantitative reverse transcriptase-polymerase chain reaction (qRT–PCR) was performed on an ABI 7500 Real-Time PCR System with 20-μl mixture according to the following procedures: 95 °C for 30 s, 40 cycles of 95 °C for 10 s, 60 °C for 30 s. Melting curves used for confirming unique amplification of each PCR reaction were conducted with the instrument’s default parameters. Three out of 15 and 17 DEGs with constitutively different expressions in the red or green *T. urticae* populations fed on cotton, cucumber and eggplant were used to qRT-PCR assays, respectively. All the qRT-PCR reactions were carried out with three biological replicates, and three samples were used in each biological replicate. The relative expression levels were computed based on the 2^-ΔΔCt^ method [101] and compared among treatments using t tests based on three biological replicates. The qPCR primers designed from Primer Premier v5.00 were listed in Table S18.

## Supplementary Information

**Additional file 1.**

**Additional file 2.**

**Additional file 3.**

**Additional file 4.**

**Additional file 5.**

**Additional file 6.**

## Data Availability

Raw data and final assembly of this project were submitted to NCBI under BioProject PRJNA577865. Raw data have been deposited in the Sequence Read Archive database of NCBI under the accession numbers of SRR10314242-SRR10314247 (genome sequence data) and SRR10313520 to SRR10313527, SRR10313531 to SRR10313542, SRR10313546 to SRR10313549, SRR13706100 to SRR13706147 (transcriptome sequence data); Whole Genome Shotgun project has been deposited at DDBJ/ENA/GenBank under the accession WHVY00000000. The files of genome assembly, annotation and CDS sequences of the red *T. urticae* were provided in Additional information (Additional files 4, 5 and 6).

## Abbreviations

CYP450s: Cytochrome P450 monooxygenases
CCEs: Carboxyl/cholinesterases
GSTs: Glutathione S-transferases
ABCs: ATP-binding cassette transporters
HGT: Horizontal gene transfer
PCA: Principal component analysis
GO: Gene Ontology
DEGs: Differentially expressed genes
qRT–PCR: Quantitative reverse transcriptase-polymerase chain reaction
UGTs: UDP-glycosyltransferases
BUSCO: Benchmarking Universal Single-Copy Orthologs
NCBI: National Center for Biotechnology Information
