# Supplementary material for: Comparative genome and transcriptome analyses reveal innate differences in response to host plants by two color forms of the two-spotted spider mite Tetranychus urticae
Source: BMC Genomics. 2021 Jul 23;22:569. doi: 10.1186/s12864-021-07894-7 (PMC8306301; doi:10.1186/s12864-021-07894-7)
Supplement: Supplementary file 1 — Additional file 1. [file 12864_2021_7894_MOESM1_ESM.zip › Figure S3.pdf]

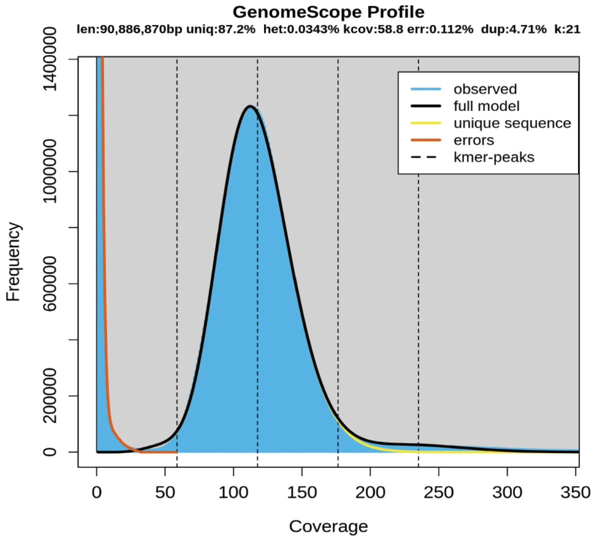
GenomeScope Profile
len:90,886,870bp uniq:87.2% het:0.0343% kcov:58.8 err:0.112% dup:4.71% k:21
observed
full model
unique sequence
errors
kmer-peaks
Frequency
0
200000
600000
1000000
1400000
0
50
100
150
200
250
300
350
Coverage

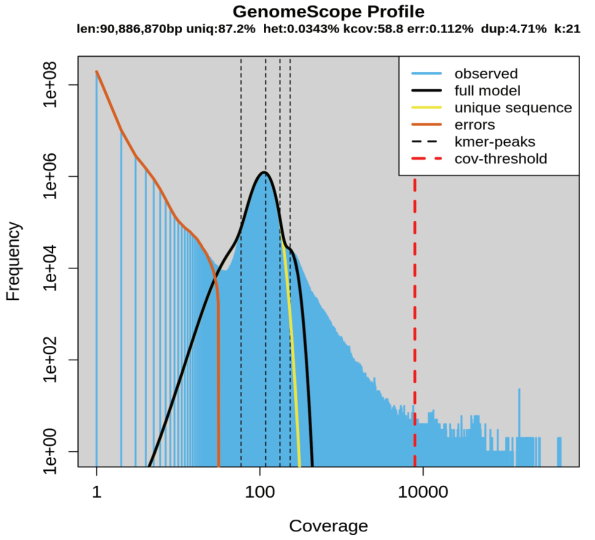
GenomeScope Profile
len:90,886,870bp uniq:87.2% het:0.0343% kcov:58.8 err:0.112% dup:4.71% k:21
observed
full model
unique sequence
errors
kmer-peaks
cov-threshold
Frequency
1e+00
1e+02
1e+04
1e+06
1e+08
1
100
10000
Coverage
