# Supplementary figures and images for: Comparative genome and transcriptome analyses reveal innate differences in response to host plants by two color forms of the two-spotted spider mite Tetranychus urticae
Source: BMC Genomics. 2021 Jul 23;22:569. doi: 10.1186/s12864-021-07894-7 (PMC8306301; doi:10.1186/s12864-021-07894-7)

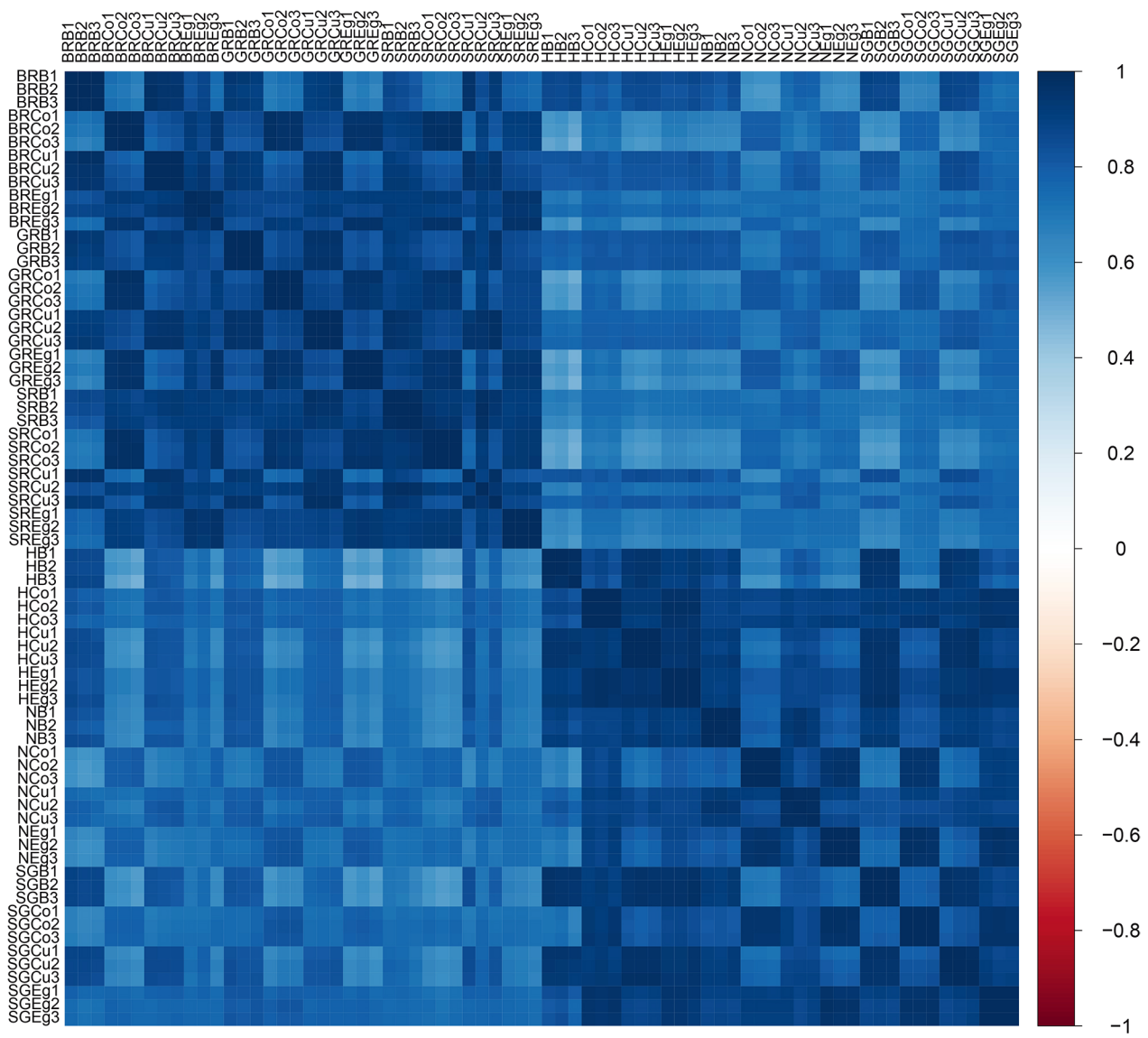

Supplement: Supplementary file 1 — Additional file 1. [file 12864_2021_7894_MOESM1_ESM.zip › Figure S1.pdf]

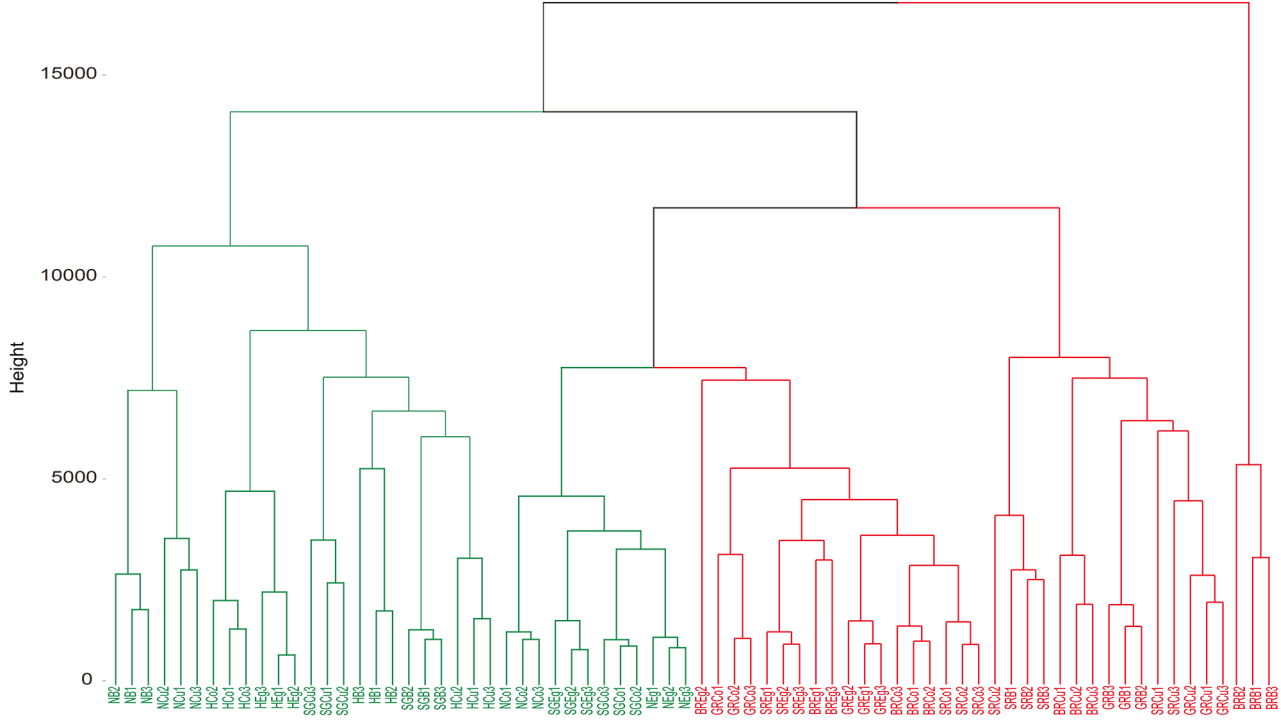

Supplement: Supplementary file 1 — Additional file 1. [file 12864_2021_7894_MOESM1_ESM.zip › Figure S2.pdf]

A

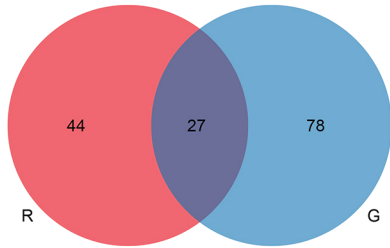

Cotton

B

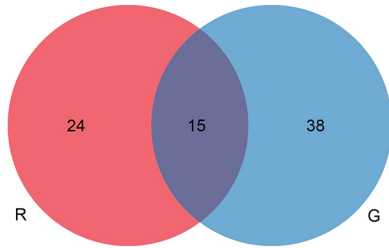

Cucumber

C

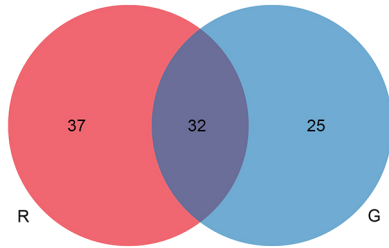

Eggplant

Supplement: Supplementary file 1 — Additional file 1. [file 12864_2021_7894_MOESM1_ESM.zip › Figure S4.pdf]

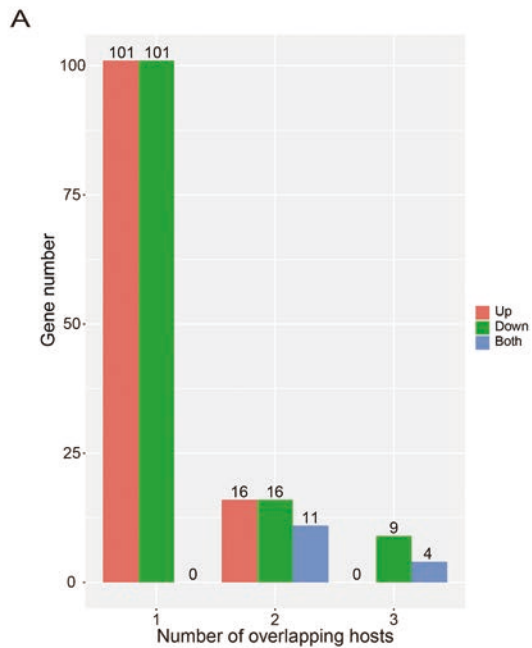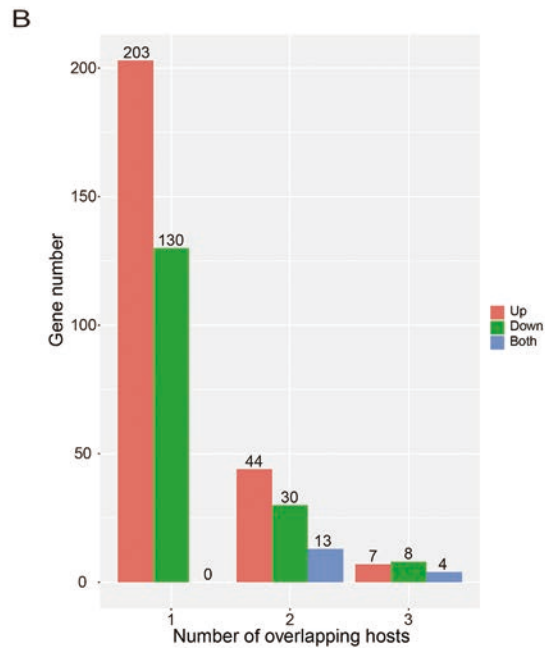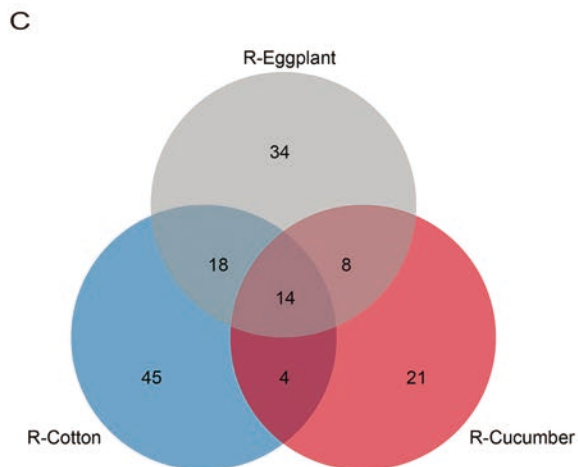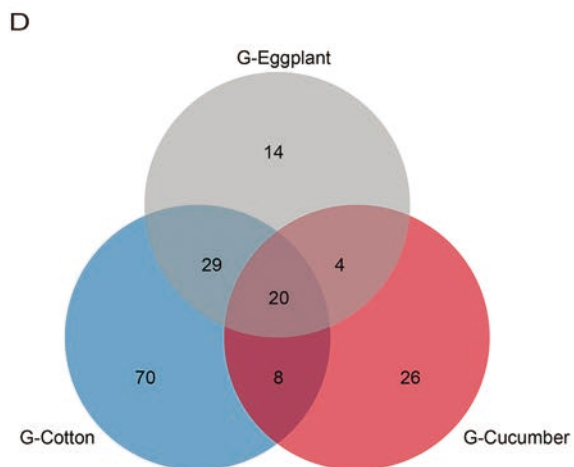

Supplement: Supplementary file 1 — Additional file 1. [file 12864_2021_7894_MOESM1_ESM.zip › Figure S5.pdf]

A

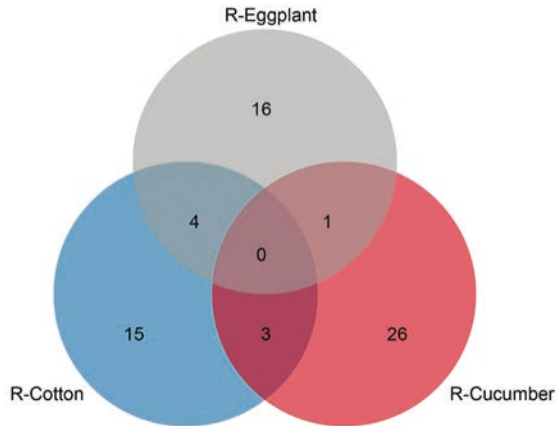

B

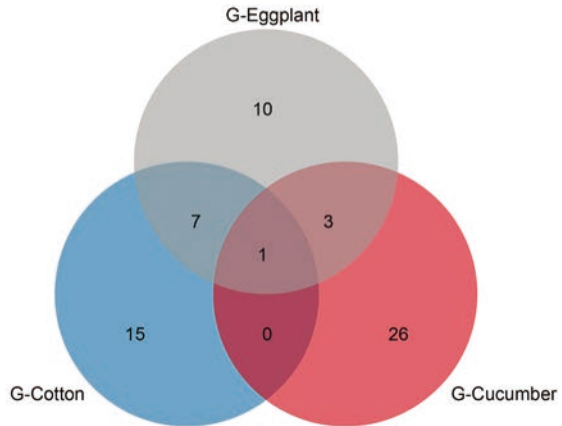

Supplement: Supplementary file 1 — Additional file 1. [file 12864_2021_7894_MOESM1_ESM.zip › Figure S6.pdf]

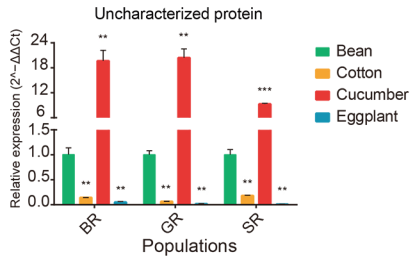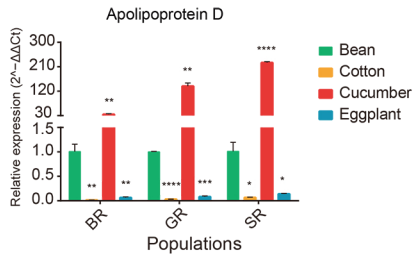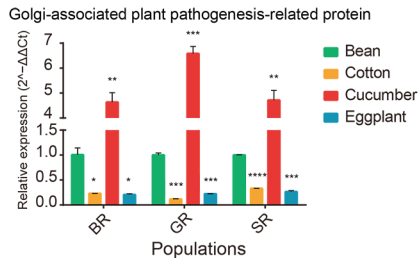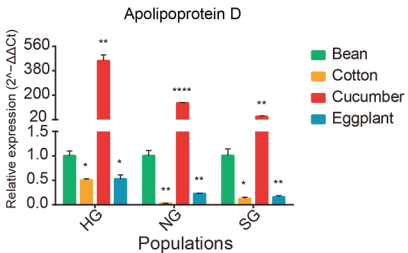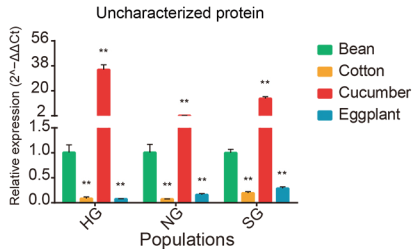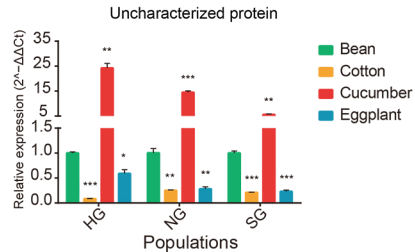

Supplement: Supplementary file 1 — Additional file 1. [file 12864_2021_7894_MOESM1_ESM.zip › Figure S7.pdf]

A

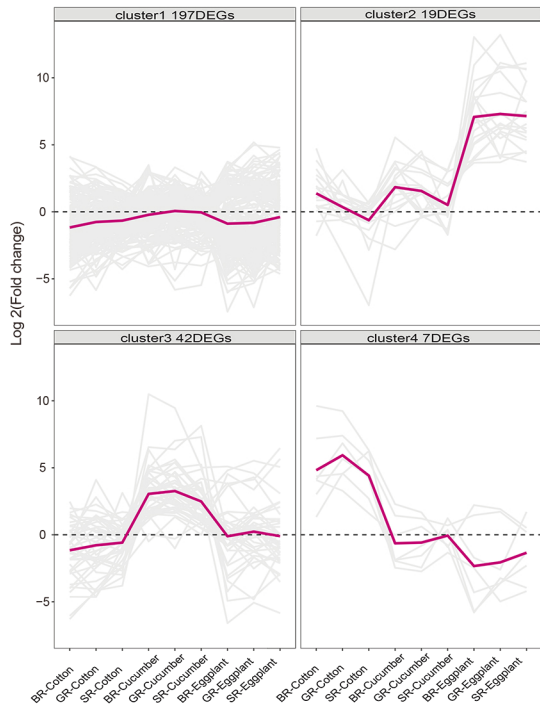

B

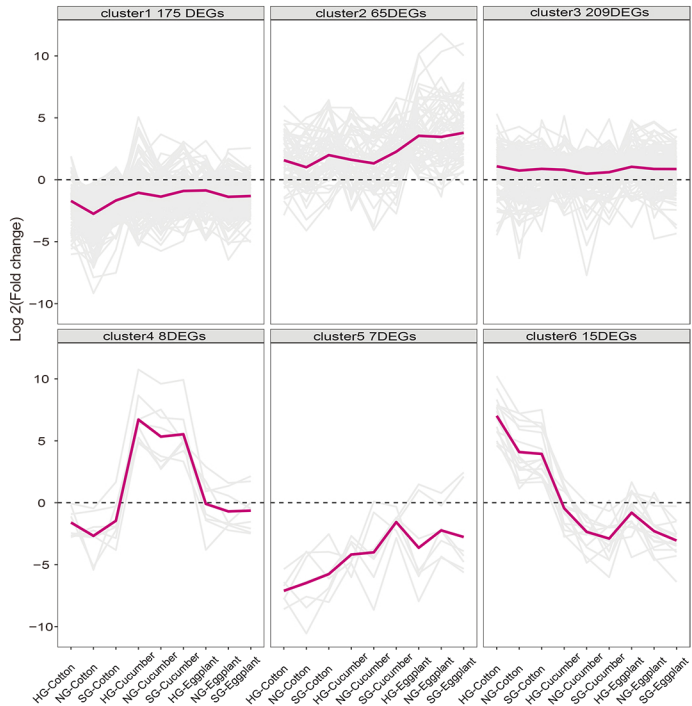

Supplement: Supplementary file 1 — Additional file 1. [file 12864_2021_7894_MOESM1_ESM.zip › Figure S8.pdf]

A

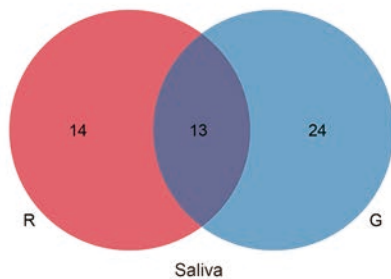

B

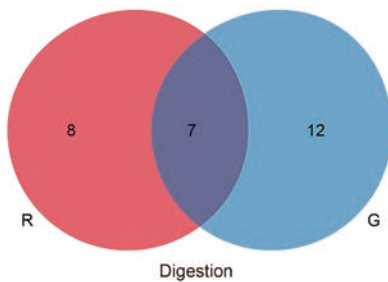

C

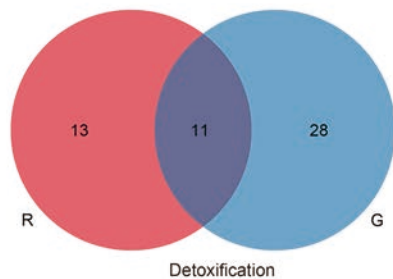

D

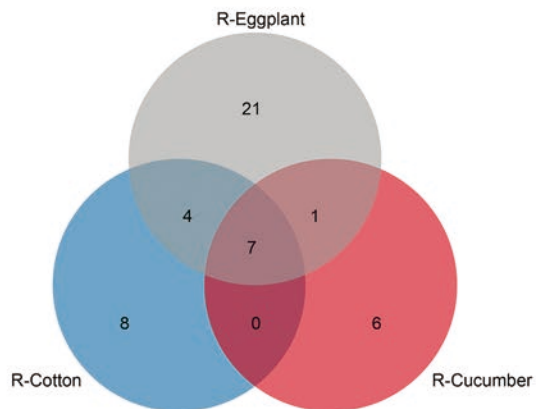

E

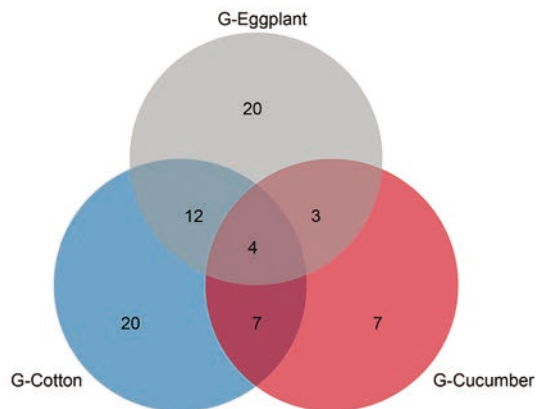

Supplement: Supplementary file 1 — Additional file 1. [file 12864_2021_7894_MOESM1_ESM.zip › Figure S9.pdf]
