# Supplementary material for: Comparative genome and transcriptome analyses reveal innate differences in response to host plants by two color forms of the two-spotted spider mite Tetranychus urticae
Source: BMC Genomics. 2021 Jul 23;22:569. doi: 10.1186/s12864-021-07894-7 (PMC8306301; doi:10.1186/s12864-021-07894-7)
Supplement: Supplementary file 3 — Additional file 3. [file 12864_2021_7894_MOESM3_ESM.doc]

**Additional Information for:**

**Comparative genome and transcriptome analyses reveal innate differences in response to host plants by two color forms of the two-spotted spider mite *Tetranychus urticae***

Shi-Mei Huo^1†^, Zhi-Chao Yan^1†^, Feng Zhang^1^, Lei Chen^1^, Jing-Tao Sun^1^, Ary A. Hoffmann^2^, Xiao-Yue Hong^1^

Correspondence:Xiao-Yue Hong, fax: +86 25 84395339; email: [xyhong@njau.edu.cn](mailto:xyhong@njau.edu.cn)

**Materials and Methods**

**Genome DNA preparation**

A red *T. urticae* strain was used for genome sequencing. The taxonomic status of the strain was determined based on morphological traits and molecular methods [1]. To reduce the effect of the major endosymbiont *Wolbachia* (which is commonly harbored in spider mites) on sequencing results, the strain was treated with tetracycline (0.1%, w/v) for three generations and maintained at a stable population density without antibiotics for eight generations before isofemale line preparation. The mites were sib mated for 10 generations, and genome DNA was isolated from adult females of the F10 generation using a Blood and Cell Culture DNA Mini kit (Qiagen, Germany) according to the manufacturer’s protocols. DNA quantity was determined by Nanodrop and gel electrophoresis.

**Genome sequencing and assembly**

High-quality genomic DNA was used to build Illumina libraries with insert sizes of 270 bp, 500 bp, and 800 bp, and two PacBio libraries with average lengths of 9 Kb. The libraries were sequenced on Illumina HiSeq X Ten and PacBio Sequel platforms (Table S3). Genome size of the red form of *T. urticae* was estimated by *k*-mer analysis. The distribution of *k*-mer frequencies was calculated with 21-mers using Jellyfish v2.2.7 [2], and genome size was estimated with GenomeScope v1.0.0 [3]. The genome assembly was performed using Flye v2.4.2 and Canu v1.3 [4, 5] with PacBio data, and two assemblies derived from Flye and Canu were further merged to improve genome contiguity with two rounds of quickmerge [6]. Heterozygous regions in the merged assembly were reduced with Purge Haplotigs v20180917 [7]. To reduce error rate resulting from PacBio sequencing, two rounds of analysis in Pilon v1.22 [8] were used to polish the assembly with Illumina reads, and we reduced redundant contigs again with Purge Haplotigs. We checked and filtered possible contaminants from humans and microbes by BlastN v2.7.1 [9] against the NCBI nucleotide database, and checked the possible contaminants with length shorter than 10,000 bp. Vector contamination was also removed using VecScreen against the UniVec database. Finally, we assessed the completeness of assemblies based on Benchmarking Universal Single-Copy Orthologs (BUSCO) [10] analyses against the arthropod dataset (n =1,066). We also mapped RNA-seq data, transcripts, Illumina raw data, and PacBio long reads to the assembly to evaluate genome completeness of the red form of *T. urticae* genome.

**Repetitive elements and non-coding RNA annotation**

Transposable elements were estimated from a combination of *de novo*- and homology-based approaches. We built a *de novo* species-specific repeat library using RepeatModeler v1.0.11 [11], and used that library combined with the RepBase-20170127 databases [12] to generate a custom library. Second, we identified repeats in the genome assembly using RepeatMasker v4.0.7 [13] with the custom library. Additionally, we identified non-coding RNAs (ncRNAs) with Infernal v1.1.2 [14] by searching for the genome of the red form of *T. urticae* in the Rfam v14.0 [15] database, and transfer RNAs (tRNAs) were further found using tRNAscan-SE v2.0 [16].

**Results**

**Genome assembly and completeness assessment**

Genome heterozygosity was estimated with GenomeScope v1.0.0 [3], and varied from 0.0304% to 0.0382% (Fig. S3). We performed completeness assessment of the red form of *T. urticae* genome with BUSCO against the arthropoda_odb9 database (n = 1,066); the results showed 92.2% complete orthologs (C) (including 87.1% single-copy orthologs (S) and 5.1% complete duplicated orthologs (D)), and 1.2% fragmented orthologs (F). Only 1.5% of conserved BUSCO groups were missing (M) from the assembly (Table 1). We also evaluated the completeness by mapping RNA-seq data to the assembly, which showed mapping rates of 96.61% (Table 1). In addition, we mapped 96.95% and 92.76% of Illumina raw data and PacBio long reads, respectively, to the assembly (Table 1).

**Annotation of repetitive elements**

Repetitive elements (12.73 Mb) accounted for approximately 14.12% of the red form of the *T. urticae* genome with the most numerous fraction of transposable elements (TEs) spanning a total length of 10.26 Mb (Table S4). Analysis of the transposable element (TE) content of the red form of the *T. urticae* genome showed a similar total proportion of TEs (11.38%) to that of the green form of *T. urticae* genome (10.01%), and DNA transposons are the most common in the red form of *T. urticae* genome (Table S4), while LTR elements are the dominant TE in the green form of *T. urticae* genome [17].

**Annotation of protein-coding gene sets and non-coding RNA**

Among the identified protein-coding genes, 9,312 (78.1%) were identified protein domains obtained from InterProScan; of these, 6,079 genes were annotated to GO terms, and 484, 400, and 2,479 genes were annotated to the KEGG, MetaCyc, and Reactome databases, respectively. ncRNAs in the red form of *T. urticae* genome were identified by Infernal v1.1.2 against the Rfam v14.0 database and by tRNAscan-SE v2.0. In total, we identified 132 rRNAs, 136 tRNAs, 22 miRNAs, 23 small nuclear RNAs, two ribozymes, and 31 other ncRNAs (Table S5).

**Additional file1_Figures**

**Figure S1: Correlation heatmaps of DEGs among the red and green *T. urticae* populations feeding on the four hosts.** Average transcript levels (FPKM) of each red and green *T. urticae* populations fed on the original host and three transferring hosts (cotton, cucumber and eggplant) were used as input for analysis. The X and Y axes represent each sample. The color represents the correlation coefficient, blue represents positive correlation and red represents negative correlation. HG (Inner Mongolia population of the green *T. urticae*); NG (Jiangsu population of the green *T. urticae*); SG (Shandong population of the green *T. urticae*); BR (Beijing population of the red *T. urticae*); GR (Guizhou population of the red *T. urticae*); SR (Shandong population of the red *T. urticae*).

**Figure S2: Hierarchical clustering of all the red and green *T. urticae* populations fed on four different hosts.** Correlation heatmap for expression of genes from the biological replicates of both forms of *T. urticae* when exposed to common bean, cotton, cucumber and eggplant. All red *T. urticae* samples are shown in red, and green *T. urticae* samples are shown in green.

**Figure S3: GenomeScope profile plots of *k*-mer frequency and log-transformed *k*-mer coverage at a *k*-mer length of 21 and a maximum *k*-mer coverage of 10,000.**

**Figure S4: Venn diagrams of DEGs between the red and green *T. urticae* feeding on the same transferring host at gene family level.** The gene families of DEGs in the red or green *T. urticae* on each of three transferring hosts: (A) cotton, (B) cucumber and (C) eggplant. The red *T. urticae* is indicated in red and the other one represents the green *T. urticae*.

**Figure S5: Summary of overlap of expression patterns of DEGs of the red and green forms of *T. urticae*, and venn diagram showing overlap among the DEG sets of the red or green form of *T. urticae* feeding on different hosts at the gene family level.** (A) and (B) show the different numbers of genes up-regulated or down-regulated or both across 1, 2, or 3 hosts. 7 and 40 DEGs in the red and green *T. urticae* are discarded due to inconsistent up- or down-regulation among the three populations of each form after the same transfer. Up-regulated DEGs are shown in red, and down-regulated DEGs are in green. Opposing expression patterns (one up-regulated and the other down-regulation in overlaps) are given in blue. (C) and (D) indicate gene family overlaps of (C) the green (D) and red forms of *T. urticae* on the different hosts.

**Figure S6: Venn diagrams of host-dependent GO terms for the red or green *T. urticae* after host transfers.** (A) represents the red *T. urticae*, and (B) represents the green *T. urticae*.

**Figure S7: Validation of transcription levels of 3 DEGs showing significantly constitutive differences in expressions among the three transfers in the red or green *T. urticae* by qPCR analysis.** The fold differences were computed using the 2^−ΔΔCt^ method. Results are mean ± SD values with three biological repeats for each gene transcript. Differences were analyzed by paired t test (*P < 0.05; **P < 0.01; ***P < 0.001; ****P < 0.0001) with GraphPad Prism v7.02 (San Diego, California USA). The data came from bean serves as control.

**Figure S8: K-means clustering of both forms of *T. urticae* DEGs upon the different host transfers.** Clusters were arranged according to the relative gene expression levels (log2 (Fold change)) of 265 and 479 DEGs in the red and green *T. urticae*, respectively. All DEGs of the red *T. urticae* responded to three transfers were categorized into four clusters, and six clusters in the green *T. urticae*. Red lines within each cluster indicate the averages expression of *T. urticae* populations upon different transfers. (A) the red form of *T. urticae*; (B) the green form of *T. urticae*.

**Figure S9:** **Venn diagrams of DEGs associated with the saliva, detoxification or digestive gene families between the two forms, and among different host populations in the red or green *T. urticae*.** (A) - (C) reflect different transcriptomic plasticity of DEGs in each family between the two *T. urticae* on (A) cotton, (B) cucumber and (C) eggplant. The red *T. urticae* is indicated in red and the blue circle represents the green form. (D) and (E) indicate the overlapping DEGs associated with saliva, detoxification and digestion of the red and green *T. urticae* on different transferring hosts, respectively.

**Additional file 2_Tables**

**Table S1.** Fecundity comparisons between two forms of *T. urticae* fed on the four host plants.

**Table S2.** Survival comparisons between two forms of *T. urticae* fed on the four host plants.

**Table S3.** Sequencing data of Illumina and PacBio libraries for the red form of *T. urticae*.

**Table S4.** Annotation of repetitive elements in the red form of *T. urticae*.

**Table S5.** Summary statistics of non-coding RNAs in the red form of *T. urticae*.

**Table S6.** Summary statistics for gene family evolution and gene count for each family and each species.

**Table S7.** Expanded and contracted gene families in each species.

**Table S8.** Rapidly evolving gene families (expansion) in the red form of *T. urticae*.

**Table S9.** GO terms of form-specific genes across three different populations of both *T. urticae* fed on three transferring hosts.

**Table S10.** List of DEGs shared by six different populations of both *T. urticae* when fed on cotton, cucumber or eggplant.

**Table S11.** List of host-specific DEGs of both *T. urticae* when fed on cotton, cucumber or eggplant.

**Table S12.** The top 10 GO terms in each category of host-specific genes for the red or green *T. urticae* fed on three transferring hosts.

**Table S13.** Constitutive differences in expressions across different populations of the red or green *T. urticae* fed on three transferring hosts.

**Table S14.** RNA-sequencing data of 6 DEGs used for qPCR of both forms of *T. urticae*.

**Table S15.** List of DEGs in the red or green *T. urticae* populations after transferring from bean to cotton, cucumber and eggplant.

**Table S16.** List of DEGs related to saliva, detoxification and digestive gene families between the two forms of *T. urticae*.

**Table S17.** List of DEGs in the saliva, detoxification and digestive gene families for the two forms of *T. urticae* fed on cotton, cucumber or eggplant.

**Table S18.** Primers used for qPCR analyses of both forms of *T. urticae*.

**Additional file 3**

**Table S19.** The file of genome assembly for the red form of *T. urticae*.

**Additional file 4**

**Table S20.** The file of genome annotation for the red form of *T. urticae*.

**Additional file 5**

**Table S21.** CDS sequence of the red form of *T. urticae* genome.

**References**

1. Seeman OD, Beard JJ. Identification of exotic pests and Australian native and naturalised species of *Tetranychus* (Acari: Tetranychidae). Zootaxa. 2011;2961(1):1-72. [http://doi.org/10.11646/zootaxa.2961.1.1](http://dx.doi.org/10.11646/zootaxa.2961.1.1)
2. Marçais G, Kingsford C. A fast, lock-free approach for efficient parallel counting of occurrences of *k*-mers. Bioinformatics. 2011;27(6):764–770. <https://doi.org/10.1093/bioinformatics/btr011>
3. Vurture GW, Sedlazeck FJ, Nattestad M, Underwood CJ, Fang H, Gurtowski J, Schatz MC. GenomeScope: Fast reference-free genome profiling from short reads. Bioinformatics. 2017;33(14):2202–2204. <https://doi.org/10.1093/bioinformatics/btx153>
4. Kolmogorov M, Yuan J, Lin Y, Pevzner PA. Assembly of long, error-prone reads using repeat graphs. Nat Biotechnol. 2019;37(5):540–546. https://doi.org/10.1038/s41587-019-0072-8
5. Koren S, Walenz BP, Berlin K, Miller JR, Bergman NH, Phillippy AM. Canu: scalable and accurate long-read assembly via adaptive *k*-mer weighting and repeat separation. Genome Res. 2017;27(5):722–736. [http://doi.org/10.1101/gr.215087.116](http://www.genome.org/cgi/doi/10.1101/gr.215087.116)
6. Chakraborty M, Baldwin-Brown JG, Long AD, Emerson JJ. Contiguous and accurate *de novo* assembly of metazoan genomes with modest long read coverage. Nucleic Acids Res. 2016;44(19):e147. <https://doi.org/10.1093/nar/gkw654>
7. Roach MJ, Schmidt SA, Borneman AR. Purge Haplotigs: allelic contig reassignment for third-gen diploid genome assemblies. BMC Bioinformatics. 2018;19:460. https://doi.org/10.1186/s12859-018-2485-7
8. Walker BJ, Abeel T, Shea T, Priest M, Abouelliel A, Sakthikumar S, Cuomo CA, Zeng Q, Wortman J, Young SK, Earl AM. Pilon: an integrated tool for comprehensive microbial variant detection and genome assembly improvement. PLoS One. 2014;9(11):e112963. <https://doi.org/10.1371/journal.pone.0112963>
9. Camacho C, Coulouris G, Avagyan V, Ma V, Papadopoulos J, Bealer K, Madden TL. BLAST+: architecture and applications. BMC Bioinformatics. 2009;10:421. <https://doi.org/10.1186/1471-2105-10-421>
10. Waterhouse RM, Seppey M, Simão FA, Manni M, Ioannidis P, Klioutchnikov G, Kriventseva EV, Zdobno EM. BUSCO applications from quality assessments to gene prediction and phylogenomics. Mol Biol Evol. 2018;35(3):543-548. <https://doi.org/10.1093/molbev/msx319>
11. Smit AFA, Hubley R. RepeatModeler Open-1.0 [cited 2018 Apr 1]. 2008-2015. Available from: [http://www.repeatmasker.org.](http://www.repeatmasker.org./)
12. Bao W, Kojima KK, Kohany O. Repbase update, a database of repetitive elements in eukaryotic genomes. Mobile DNA. 2015;6:11. https://doi.org/10.1186/s13100-015-0041-9
13. Smit AFA, Hubley R, Green P. RepeatMasker Open-4.0 [cited 2018 Apr 1]. 2013-2015. Available from: [http://www.repeatmasker.org.](http://www.repeatmasker.org./)
14. Nawrocki EP, Eddy SR. Infernal 1.1: 100-fold faster RNA homology searches. Bioinformatics. 2013;29(22): 2933–2935. <https://doi.org/10.1093/bioinformatics/btt509>
15. Kalvari I, Argasinska J, Quinones-Olvera N, Nawrocki EP, Rivas E, Eddy SR, Bateman A, Finn RD, Petrov AI. Rfam 13.0: shifting to a genome-centric resource for non-coding RNA families. Nucleic Acids Res. 2018;46(D1):D335–D342. <https://doi.org/10.1093/nar/gkx1038>
16. Lowe TM, Eddy SR. tRNAscan-SE: a program for improved detection of transfer RNA genes in genomic sequence. Nucleic Acids Res. 1997;25(5), 955–964. <https://doi.org/10.1093/nar/25.5.955>
17. Grbić M, Van Leeuwen T, Clark RM, Rombauts S, Rouzé P, Grbić V, [Osborne](https://www.nature.com/articles/javascript:;) EJ, [Dermauw](https://www.nature.com/articles/javascript:;) W, [Ngoc](https://www.nature.com/articles/javascript:;) PCT, [Ortego](https://www.nature.com/articles/javascript:;) F, et al. The genome of *Tetranychus urticae* reveals herbivorous pest adaptations. Nature. 2011;479(7374):487-492. <https://doi.org/10.1038/nature10640>
